# Differential Gene Expression Profiling of Enriched Human Spermatogonia after Short- and Long-Term Culture

**DOI:** 10.1155/2014/138350

**Published:** 2014-03-12

**Authors:** Sabine Conrad, Hossein Azizi, Maryam Hatami, Mikael Kubista, Michael Bonin, Jörg Hennenlotter, Markus Renninger, Thomas Skutella

**Affiliations:** ^1^Institute of Anatomy, University of Tübingen, Österbergstraße 3, 72074 Tübingen, Germany; ^2^Institute for Anatomy and Cell Biology, Medical Faculty, University of Heidelberg, Im Neuenheimer Feld 307, 69120 Heidelberg, Germany; ^3^Amol University of Special Modern Technologies, Special Modern Technologies, P.O. Box 46168-49767, Amol, Iran; ^4^Department of Stem Cells and Developmental Biology, Royan Institute, P.O. Box 19395-4644, Tehran, Iran; ^5^TATAA Biocenter AB, Odinsgatan 28, 41103 Göteborg, Sweden and Institute of Biotechnology at the Czech Academy of Sciences, Vídenská 1083, 14220 Prague 4, Czech Republic; ^6^Institute of Anthropology and Human Genetics, Microarray Facility, University Clinic, Calwerstraße 7, 72076 Tübingen, Germany; ^7^Department of Urology, University Clinic Tübingen, Hoppe-Seyler-Straße 3, 72076 Tübingen, Germany

## Abstract

This study aimed to provide a molecular signature for enriched adult human stem/progenitor spermatogonia during short-term (<2 weeks) and long-term culture (up to more than 14 months) in comparison to human testicular fibroblasts and human embryonic stem cells. Human spermatogonia were isolated by CD49f magnetic activated cell sorting and collagen^−^/laminin^+^ matrix binding from primary testis cultures obtained from ten adult men. For transcriptomic analysis, single spermatogonia-like cells were collected based on their morphology and dimensions using a micromanipulation system from the enriched germ cell cultures. Immunocytochemical, RT-PCR and microarray analyses revealed that the analyzed populations of cells were distinct at the molecular level. The germ- and pluripotency-associated genes and genes of differentiation/spermatogenesis pathway were highly expressed in enriched short-term cultured spermatogonia. After long-term culture, a proportion of cells retained and aggravated the “spermatogonial” gene expression profile with the expression of germ and pluripotency-associated genes, while in the majority of long-term cultured cells this molecular profile, typical for the differentiation pathway, was reduced and more genes related to the extracellular matrix production and attachment were expressed. The approach we provide here to study the molecular status of *in vitro* cultured spermatogonia may be important to optimize the culture conditions and to evaluate the germ cell plasticity in the future.

## 1. Introduction

In humans the process of spermatogenesis is initiated from a small pool of self-renewing stem cells quite late at puberty (10–13 years after birth) and continues throughout life. Human spermatogonial stem cells (hSSCs) have been for the first time identified by Clermont [1]. These cells are positioned in a developmental cascade originating from the embryonic epiblast during gastrulation, followed by primordial germ cells (PGCs) and gonocytes. Although still a difficult task, the newly established enrichment and* in vitro* propagation of spermatogonia that carry the male genome from generation to generation provide an important step not only for germ cell biology, but also for future transplantation and restoration of fertility in the clinic [2]. Recently, Sadri-Ardekani et al. [3] provided evidence for a potential clinical application by the* in vitro* propagation of prepubertal and adult hSSCs. Furthermore understanding the molecular mechanisms of hSSCs in relation to germ cell cancer development is of massive clinical importance [4].

The strategy of the isolation and short-term cultivation of spermatogonia is in our hands a prerequisite for the generation of pluripotency of these unipotent adult stem cells* in vitro* [5]. The separation of human spermatogonial stem/progenitor cells has been achieved by our group with magnetic activated cell sorting (MACS), using the antibody to CD49f (integrin alpha-6) followed by matrix selection (collagen nonbinding, laminin binding) to enrich the SSCs from human testis. Several groups successfully established in parallel similar techniques and improved approaches to enrich and culture spermatogonia even for longer time periods [6–11].

Since it is now possible to isolate and culture spermatogonia, there is major interest to understand the self-renewal and germ-associated networks of human adult SSCs and to improve the culture conditions in terms of their stemness and plasticity. It is of upmost importance to show the germ origin of these human testis-derived stem cells that spontaneously behave like pluripotent ESC-like cells that can differentiate into a number of cell lineages comprising the three embryonic germ layers [5, 9, 12–14]. In spite of different approaches in most studies only spermatogonia-enriched cell populations and consequently heterogeneous cell cultures were retrieved, which might mimic the real character and molecular status of spermatogonia during culture* in vitro*. In the studies by Mizrak et al., Chikhovskaya et al., and Gonzalez et al., the cells expressing markers of pluripotency were more mesenchymal stem cell (MSC)-like or probably derived from MSCs [14–16]. In contrast Stimpfel et al. [17] could show that both germ- and mesenchyme-derived cells were present in stem cell clusters from human testis biopsies, which could differentiate in all three germ layers* in vitro* and Lim et al. [18] demonstrated* in vitro* culture-induced pluripotency of hSSCs including teratoma formation. Furthermore renal [19] and hepatic differentiation of hSSCs [20] was observed.

One main step in analyzing the biology of SSCs is to determine their germ cell-specific gene expression profile. The present knowledge regarding the molecular markers that define hSSCs is still significantly limited [21]. The rarity of human testicular tissue available for research, the relatively low number of adult stem cells in the testis, the heterogeneity of human testis tissue available for research, the lack of unique surface markers, and the absence of a robust proliferative* in vitro* culture system to support their self-renewal have prevented so far the efficient isolation and culture of SSCs with high purity for further study. Therefore, the aim of this study was to provide evidence for molecular signatures of human spermatogonia in germ cell cultures, both after short- and long-term culture* in vitro*. In order to accomplish this goal we sought to compare the gene expression profiles of CD49f-positive/matrix enriched human spermatogonia, human testicular fibroblasts (htFibs), and hESCs using microarray analysis and nanofluid real-time PCR (Fluidigm). To get as much a real picture as possible and to avoid the bias because of testicular cell heterogeneity, the cells to be analyzed were individually selected from the spermatogonia-enriched populations cultured* in vitro* by a micromanipulation system.

## 2. Results

### 2.1. Selection and Culture of Human Spermatogonia

**Figure 1:**
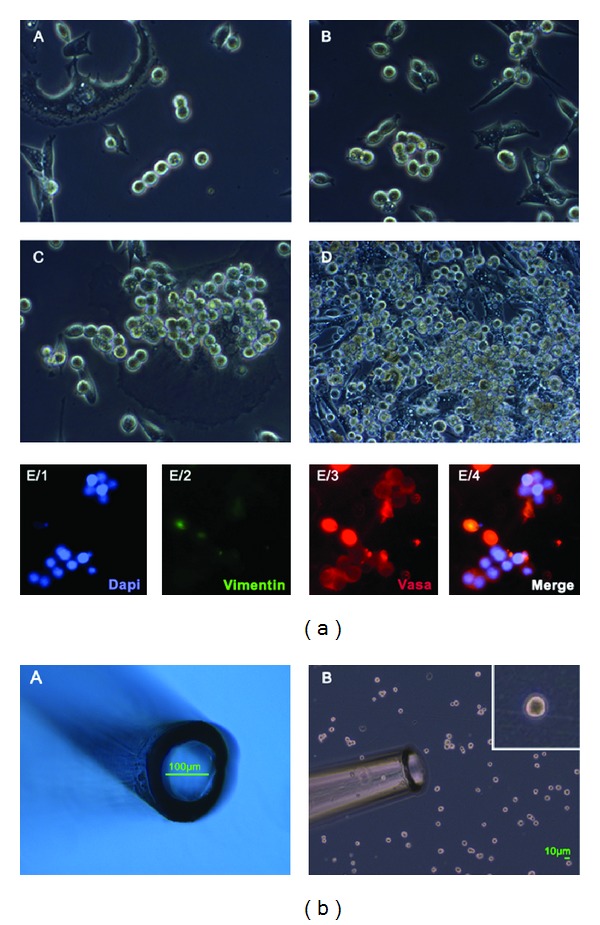
Human spermatogonia cultured* in vitro* after CD49f- and matrix-selection. (a) Typical morphology of spermatogonia from same patient 184 during culture: (A) after one week, (B) after one month, (C) after three months, and (D) after six months. Single and interconnected round cells with high nucleus/cytoplasm ratio typical of spermatogonia were observable. Pairs, chains, and small groups/colonies of interconnected spermatogonia were present in all cell cultures. Long-term cultured cells were grown on inactivated CF1 feeder cells. Cultures were devoid of human fibroblasts. (E) Purified VASA-positive germ cell cultures were devoid of VIMENTIN-positive somatic cells. (b) Collection of spermatogonia by a micromanipulation system (micropipette) for gene expression profiling (A). (B) Example of selected spermatogonia is shown as insert. Magnification (a): (A)–(D) 10x, (E/1)–(E/4) 20x; (b): (B) 5x, insert 20x.

**Figure 2:**
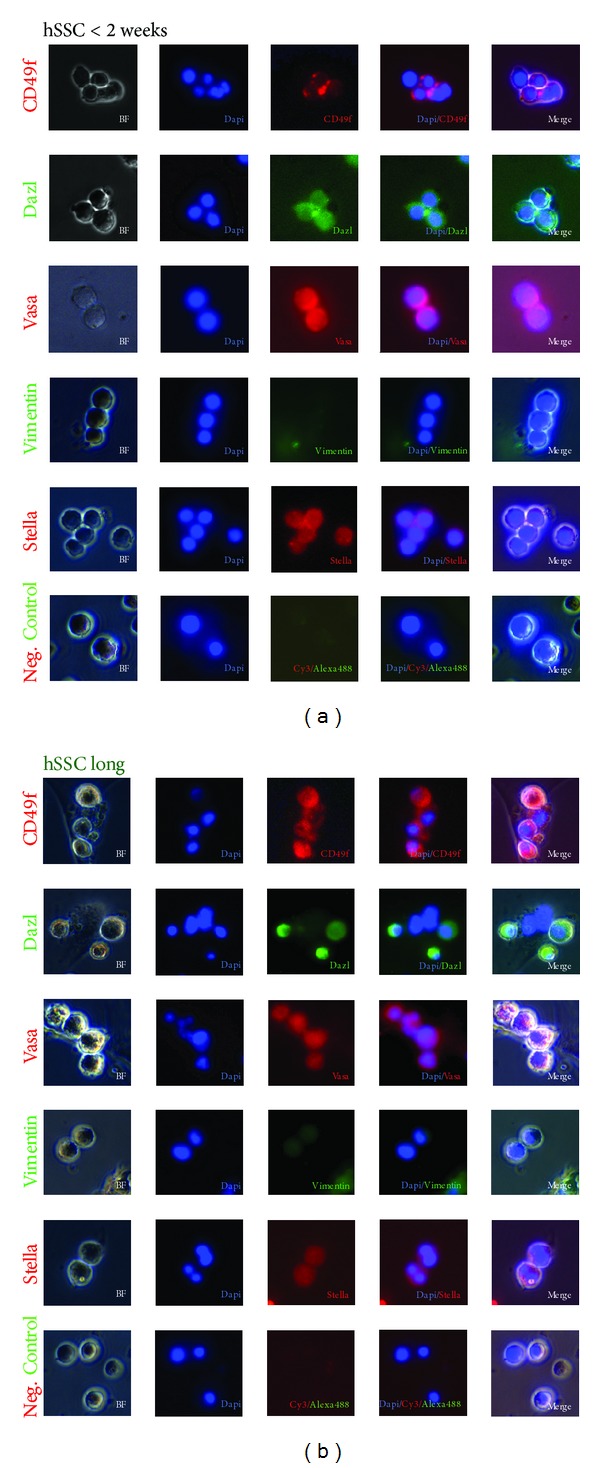
Immunohistochemical analysis of (a) hSSC < 2 weeks.Cells were stained with CD49f, DAZL, VASA, VIMENTIN, and STELLA. For VIMENTIN the cultures were negative. Magnification 20x. Immunohistochemical analysis of (b) hSSC long-term cultures. Cells were stained with CD49f, DAZL, VASA, VIMENTIN, and STELLA. For VIMENTIN the cultures were weakly positive. Magnification 20x.

The spermatogonia were enriched using CD49f-MACS and matrix selection (collagen nonbinding/laminin binding) from orchiectomies (see Tables S1 and S2 (in Supplementary Material available online at http://dx.doi.org/10.1155/2014/138350) for patient information related to the SSC cultures). In the selected populations of cells spermatogonia may be the predominating cells, as revealed by positive DDX4 (VASA) and negative VIMENTIN immunocytochemistry of early cultures (Figures 1 and 2), while long-term cultured cells showed reduced VASA staining and were weakly VIMENTIN-positive (Figure 2(b)). The morphology of purified spermatogonia isolated from the different patients was similar irrespective of the age of the patients and days of culture. This was primarily based on their round shape morphology and size of approximately 6–12 *μ*m in diameter, high nucleus-to-cytoplasm ratio, which could be observed by a clear small shining cytoplasmic ring between the round nucleus and the outer cell membrane. Pairs, chains, and small groups of spermatogonia interconnected by intercellular bridges were present in all cell cultures. In the cultures also some other types of cells, for example, bigger cells with a diameter of 12–14 *μ*m, an ovoid shape, and a low nucleus/cytoplasm ratio, were observed. htFibs were depleted and mostly remained in the nonselected populations of cells. The htFibs, which easily overgrew the primary cell cultures, were successfully derived from the nonselected cell fractions [18]. Representative examples of spermatogonial cultures devoid of htFibs are shown in Figure 1.

### 2.2. Pilot Study of Gene Expression Profiling of Enriched Human Spermatogonia after Short-Term and Long-Term Culture with Nanofluidic Real-Time PCR

**Figure 3:**
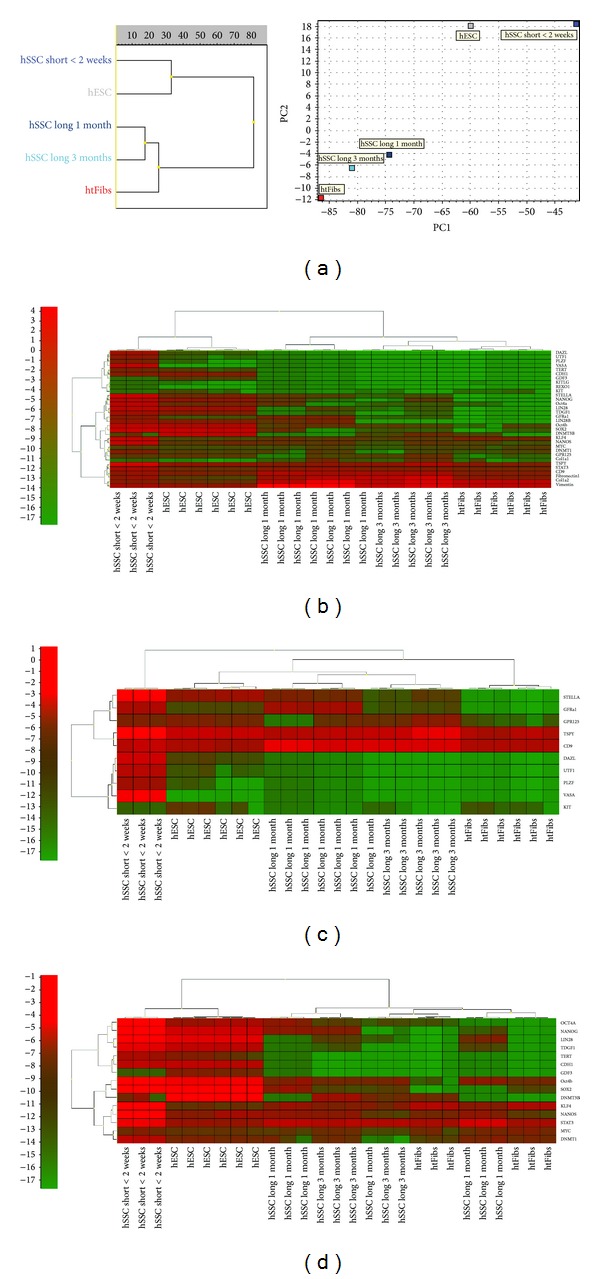
Germ-, pluripotency-, and fibroblast-related gene expression profiling of samples with 50 spermatogonia from short-term (patient 195) and long-term cultures (patients 184, 194) in comparison with human embryonic stem cells and human testis fibroblasts with Fluidigm BioMark system. (a) Dendogram and PCA. Heatmaps ranging from strongly expressed (red) to absent (green) with hierarchical clustering are shown (b) for all analysed genes, (c) for germ- and (d) for pluripotency-associated genes.

The nanofluidic real-time PCR gene expression profiling of samples with 50 spermatogonia collected by micromanipulator from short-term culture in one patient (patient 195; duration of culture <2 weeks) and long-term cultures in two patients (patients 184 and 194; duration of cultures between 1 and 6 months) revealed that these cells had a typical germ cell profile (Figure 3 and Figure S1). When analysing all the selected germ-, pluripotency-, and fibroblast-associated genes, profiling showed that short-term cultured spermatogonia separated from all the other groups of cells and were closely related to hESCs in the same dendrogram subtree, while long-term cultured spermatogonia, although positioned in their own subtree, were closer related to htFibs with increasing duration of the culture (Figure 3(a)). This depended on overall changes in the expression of the germ- and pluripotency-associated genes but also related to an increase in more genes related to the extracellular matrix production and attachment during prolonged germ cell cultivation.

The short-term cultured spermatogonia expressed a high level of the known germ cell-specific genes* DDX4 *(*VASA*),* DAZL*,* DPPA3 *(*STELLA*),* ZBTB16* (*PLZF*), and the GDNF-receptor GFR*α*-1, while being negative for* c-KIT* (short-term spermatogonia <2 weeks versus htFibs, Mann-Whitney test, *P* < 0.05) (Figure 3(b) and Figure S1a). Furthermore these cells highly expressed the pluripotency genes* LIN28*,* NANOG*,* TDGF1*,* CDH1*,* TERT,* and POU5F1 (*OCT4A)*.

In sharp contrast, the htFibs did not express 15 out of the 32 germ- and pluripotency-associated genes, namely,* DDX4 *(*VASA*)* DAZL*,* DPPA3* (*STELLA*),* GFRA1*,* PLZF*,* LIN28*,* GDF3*,* NANOG*,* UTF1*,* TDGF1*,* DNMT3B*,* LIN28B*,* TERT*,* CDH1*, and* SOX2,* at all and were positive for* c-KIT*.

Long-term cultured spermatogonia showed reduced expression of* DDX4* (*VASA*),* DAZL*, and* ZBTB16* (*PLZF*) but stayed positive for the germ cell markers* DPPA3* (*STELLA*) and GFR*α*-1, which were not expressed in fibroblasts (long-term spermatogonia versus htFibs, Mann-Whitney test, *P* < 0.05). Furthermore, the germ and pluripotency cell markers* NANOG*,* LIN28*,* LIN28B,* and* TDGF1 *also showed this all-or-none expression pattern as seen in the heat-map for short- and long-term cultured spermatogonia but were not expressed in htFibs (short- and long-term spermatogonia versus htFibs, Mann-Whitney test, *P* < 0.05) (Figure 3(b) and Figure S1a). In comparison to htFibs long-term cultured spermatogonia also significantly expressed, although to a lower extent, the germ cell- and pluripotency-related genes* CD9*,* STAT3*,* TDGF1*,* TSPYI*,* OCT4A,* and* NANOS*, while htFibs significantly overexpressed the genes* KIT* and* MYC *in comparison to long-term cultured spermatogonia (long-term spermatogonia <2 weeks versus htFibs, Mann-Whitney test, *P* < 0.05). The pluripotency associated genes* SOX2*,* DNMT3B*,* OCT4A*, LIN28, KLF4, S*TAT3,* and* MYC *were expressed in all long-term cultures of spermatogonia.

The genes* COL1A1*,* FIBRONECTIN1,* and* VIMENTIN*, which were weakly expressed in short-term cultured spermatogonia, were upregulated in long-term cultured spermatogonia (long-term versus short-term spermatogonia <2 weeks, Mann-Whitney test, *P* < 0.05) (Figure 3(b) and Figure S1a). This indicates that long-term cultured spermatogonia expressed more extracellular matrix molecules during prolonged culture, while retaining a germ and pluripotency profile, although the expression of these genes decreased during culture up to 6 months.

As can be seen in the PCAs in Figure S1a, there was a clear difference in the expression of germ and pluripotency genes in short- and long-term cultured spermatogonia and htFibs, although, in the case of long-term cultured spermatogonia, the number of differentially expressed germ cell-associated genes was lower. On the other hand, it became obvious that a part of the genes* COL1A1*,* FIBRONECTIN1,* and* VIMENTIN* shifted over to the side of the long-term cultured spermatogonia (Figure S1b).

The real-time PCR data was further supported by immunocytochemical analysis (Figures 2(a) and 2(b)). Short-term cultured spermatogonia stained positive for CD49f, VASA, DAZL, and STELLA and were negative for VIMENTIN. Long term-cultured cells showed weaker signals for CD49f, VASA, DAZl, and STELLA and stained slightly positive for VIMENTIN.

These observations encouraged us to have a closer look at the changes in genes' global expression and in particular the expression of germ-, pluripotency-, and fibroblast-associated genes and genes activated during the short- and long-term culturing of human spermatogonia.

### 2.3. Microarray Gene Expression Profiling of Human Spermatogonia

#### 2.3.1. Dendrograms of Sample Relations

**Figure 4:**
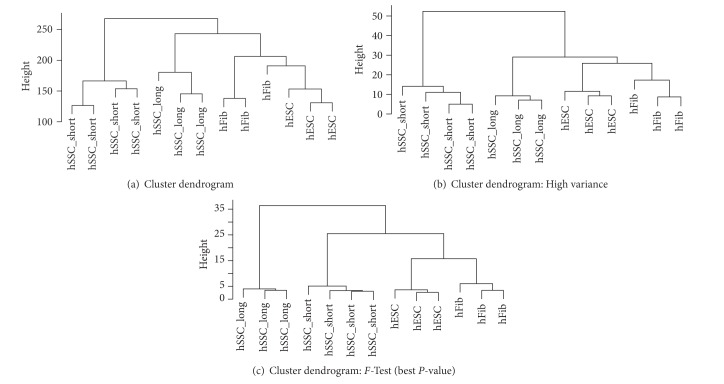
Genome-wide profiling of spermatogonia in comparison with htFibs and hESCs. (a) Dendrogram based on the whole transcriptome, calculated using complete linkage clustering and Euclidean distance. Samples were grouped almost perfectly according to their cell type. Short-term cultured spermatogonia (spermatogonia_short, left hand side of the tree) were poorly related to other cell types. hESCs have a rather high degree of relationship to fibroblasts. (b) Dendrogram based on the 150 features with highest variances, calculated using complete linkage clustering and Euclidean distance. All cell types were grouped correctly into subtrees. (c) Dendrogram based on the 150 features with highest discrimination power assessed by the *P* value of an* F*-test. Using a set of markers designed for distinguishing the groups, separation of the cell types was even stronger in comparison with (a) and (b). The overall topology remains very similar in all cases.

The dendrograms of sample relations display the distances or degree of relationship between experimental samples (Figure 4). Three different dendrograms were calculated, comparing germline-derived cells: short- and long-term cultured hSSCs to somatic cells: htFibs and hESCs. All dendrograms were created using hierarchical complete linkage clustering with Euclidean distance. The three dendrograms were calculated: (1) using the whole transcriptome assessed by the Affymetrix platform (Figure 4(a)), (2) considering only 150 features with highest variances (Figure 4(b)), and (3) using the 150 features with highest discrimination power assessed by the *P* value of an* F*-test (Figure 4(c)).

On the level of whole transcriptome, hSSC short- and long-term cultures cells were clearly separated into different subtrees (Figure 4(a)). In particular short-term cultured spermatogonia were substantially different from the other cell types. hESCs had their highest degree of relationship to fibroblasts being located in the same subtree. One of the htFibs samples seems to be more closely related to hESCs. Looking at the dendrogram, calculated from the 150 high variance features (Figure 4(b)), the separation of the cell types became even more evident and all cell types were grouped correctly into subtrees. Again, the hSSC short-term cultured cells were very distinct from the other cell types. The previously outlying htFibs sample was now similar to the other htFibs. The separation of the cell types was even stronger using the top 150 features with most significant *P* values of an* F*-test (Figure 4(c)).

This indicates that short-term cultured spermatogonia from one patient join at similar levels, as do long-term cultured spermatogonia from two patients, and also htFibs and hESCs. All cell types have their distinct expression pattern and they are clearly distinguishable from each other. This was true for the selected set of genes based on high variance genes but also visible using the complete transcriptome (Figure 4).

#### 2.3.2. Differential Analysis of Short- and Long-Term Cultured Spermatogonia in Comparison with Somatic htFibs and hESCs

**Figure 5:**
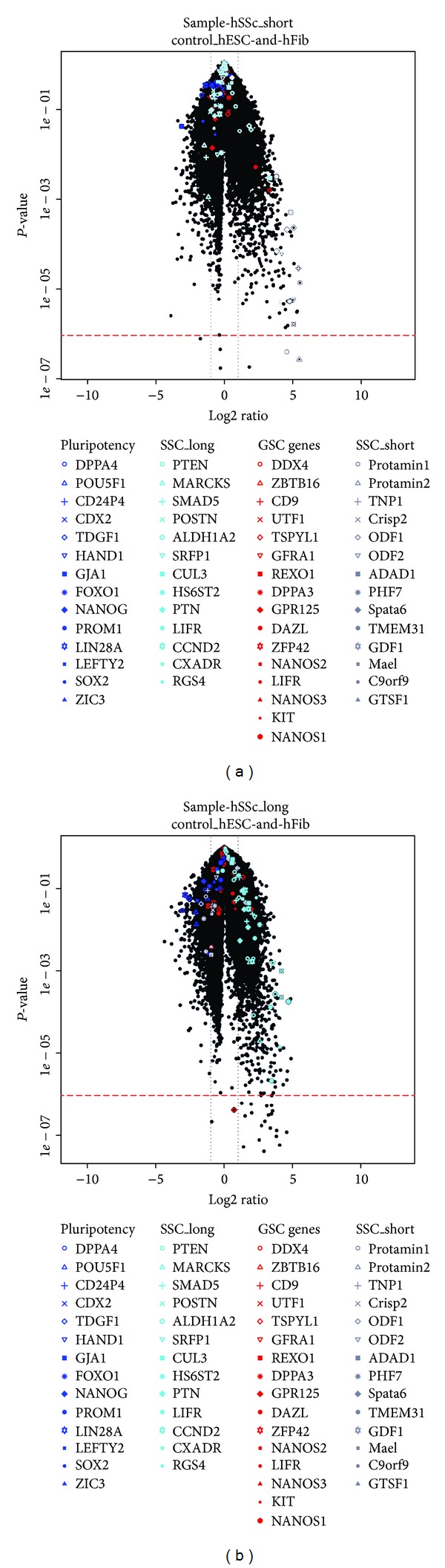
Volcano plots (*x*-axis: log2 ratio and *y*-axis:* t*-test *P* value). (a) SSC_short (short-term cultured spermatogonia) versus hESCs and htFibs. (b) SSC_long (long-term cultured spermatogonia) versus hESCs and htFibs. Expression of four different sets of genes (pluripotency-, GS genes, SSC_short-, and SSC_long-associated genes) were analysed.

Differential analysis identified genes up- or downregulated in the two sets of samples. Standard* t*-test comparing groups were performed. Figures 5 and S2 show volcano plots visualizing fold changes and* t*-test *P* values of the comparisons of the short-term and long-term cultured spermatogonia with the control group composed of htFibs and hESCs (Figure 5). For both analyses genes showed strong fold changes up to a 32-fold differential regulation (log2 folds up to 5).

Differential analyses were used to define lists of germ cell-associated genes upregulated in short- (SSC_short:* PROTAMIN 1*,* PROTAMIN 2*,* TNP1*,* CRISP2*,* ODF1*,* ODF2*,* ADAD1*,* PHF7*,* SPATA6*,* TMEM31*,* GSF1*,* MAEL*,* C9ORF9*, and* GTSF1*) and long-term cultured spermatogonia (SSC_long:* PTN*,* MARCKS*,* SMAD5*,* POSTN*,* ALDH1A2*,* SRFP1*,* CUL3*,* HS6ST2*,* PTEN*,* LIFR*,* CCND2*,* CXADR*, and* RGS4*) when compared to testis htFibs and hESCs.

Additional sets of literature-derived pluripotency-associated genes (pluripotency:* POU5F1 (OCT4)*,* LEFTY2*,* CDX2*,* FOXO1*,* LIN28A*,* HAND1*,* DPPA4*,* SOX2*,* ZIC3*,* GJA1*, and* NANOG*) and germ cell-enriched genes (GS genes:* TSPY1*,* DDX4* (*VASA*),* UTF1*,* CD9*,* ZBTB16* (*PLZF*),* TSPYL1*,* GPR125*,* DAZL*,* GFR*α*-1*,* DPPA3* (*STELLA*),* NANOS2*,* NANOS1*,* NANOS3*,* KIT*, and* LIFR*) were defined. Genes in these lists are highlighted in the volcano plots.

Next we focused on these gene sets and visualized gene expression in a heat map (Figure S3). The corresponding volcano plots are provided as supplemental material (Figure S2), with tables showing the 20 most strongly expressed genes and the 20 most upregulated stem (pluripotency) and germ-cell-associated genes (Table S3).

### 2.4. Control Cells

When comparing control htFibs and hESCs based on the selected gene lists it became clear that, as expected, pluripotency-associated genes* POU5F1 *(*OCT4*),* SOX2*,* NANOG*,* LEFTY2 *and* DPPA4*,* GJA1,* and* LIN28A *were higher expressed in hESCs (log2 fold up to 5.2), as seen in Figure S3A. Interestingly, also some of the germ cell-associated genes:* SRFP1*,* PTN*,* CXADR*,* CCND2*,* ALDH1A2* (from SSC_long list),* SPATA6*,* PROTAMIN1* (*PRM1*),* TNP1*,* ODF1, *and* ODF2* (from SSC_short list) were higher expressed in hESCs compared to htFibs. In the comparison of htFibs and hESCs, among the 25 genes listed above for short- and long-term cultured spermatogonia (SSC_short and SSC_long genes) only* POSTN* is differentially overexpressed in htFibs by more than log2 fold >2.

#### 2.4.1. Short-Term Cultured Spermatogonia

When comparing short-term cultured spermatogonia with htFibs and hESCs, the established germ cell markers* DDX4* (*VASA*),* TSPY1,* and* DAZL* (GS genes), which were mostly expressed in germ stem and progenitor cells, were upregulated in spermatogonia (log2 folds up to 3.3), as can be seen in Figure 5 and Figures S3 and S4. In addition,* TSPY3* was also strongly upregulated. Other known germ cell markers were not differentially expressed. ES-specific genes* POU5F1*,* NANOG*,* LIN28,* and* LEFTY2* showed high expression in hESCs but low expression in short-term cultured spermatogonia. The most striking observation was that many upregulated genes (with log2 folds up to 5.5) were known to be related to germ cells. The most upregulated genes were associated with the germ cell differentiation pathway and include genes important for spermatogenesis, such as* PROTAMIN2* and* PROTAMIN1* (*PRM2*,* PRM1*),* TMEM31*,* CRISP2*,* PHF7*,* ODF2*,* ADAD1*,* TNP1,* and* SPATA6 *(see also volcano plot in Figure 4). The germ cell-associated genes* CUL3*,* ALDH1A2,* and* PTEN *from SSC_long gene list were also upregulated in the short-term cultured cells as well (log2 folds up to 3.3). It has to be pointed out that most of the germ cell-associated genes (GSC genes) and those highly upregulated in short-term cultured spermatogonia (SSC_short genes) had low expression in htFibs and hESCs. It also should be noted that several genes, classified as typical spermatogonial markers, such as* ZBTB16* (*PLZF*), GFR*α*-1, and* DPPA3 *(*STELLA*) were not found to be highly expressed on the Affymetrix chips.

#### 2.4.2. Long-Term Cultured Spermatogonia

In comparison of long-term cultured spermatogonia with htFibs and hESCs, no pluripotency-associated genes were upregulated in these cells (Figure 5 and Figures S3 and S4). However,* GPR125*,* LIFR*,* KIT,* and* KITLG from the known germ cell markers *(GSC genes) showed the highest expression in long-term cultured spermatogonia. Only a single probe for* SPATA6* gene from the list of short-term cultured spermatogonia (SSC_short genes) was upregulated with a log2 fold of 1.5. A number of the highly expressed markers in long-term cultured SSCs (SSC_long genes) were stem- and germ cell-related genes. The most upregulated genes in this group were* HS6ST2*,* POSTN*,* SRFP1*,* ALDH1A2*,* LIFR*,* CCND2*,* RGS4*,* MARCKS*,* PTEN*, and* PTN*. Several genes from the SSC_long list were highly expressed also in hESCs (*MARCKS*,* PTEN*,* CCND2*,* PTN*,* PTEN*,* SFRP1,* and* CUL3*) but not in fibroblasts.

#### 2.4.3. Differences between Long-Term and Short-Term Cultured Spermatogonia

When comparing short- and long-term cultured spermatogonia directly with each other, short-term cultured spermatogonia showed an upregulation of the pluripotency-associated genes* POU5F1 *(*OCT4*),* LEFTY2,* and* DPPA4* (log2 folds 1.5) and of the germ cell-associated genes* DDX4 *(*VASA*) (log2 fold 4.25),* TSPY1* (log2 fold 3.8), and* DAZL* (2.66) (Figure S2). Most of the short-term SSC-associated genes (SSC_short genes) showed rather low expression in long-term cultured spermatogonia. Some genes from the SSC_long gene list also showed high expression in short-term cultured spermatogonia (i.e.,* PTEN* and* CUL3*).

#### 2.4.4. Analysis of Short- and Long-Term Cultured Spermatogonia with Predefined Gene Sets for Germ-, Pluripotency-, and Fibroblast-Associated Genes from the Literature

**Figure 6:**
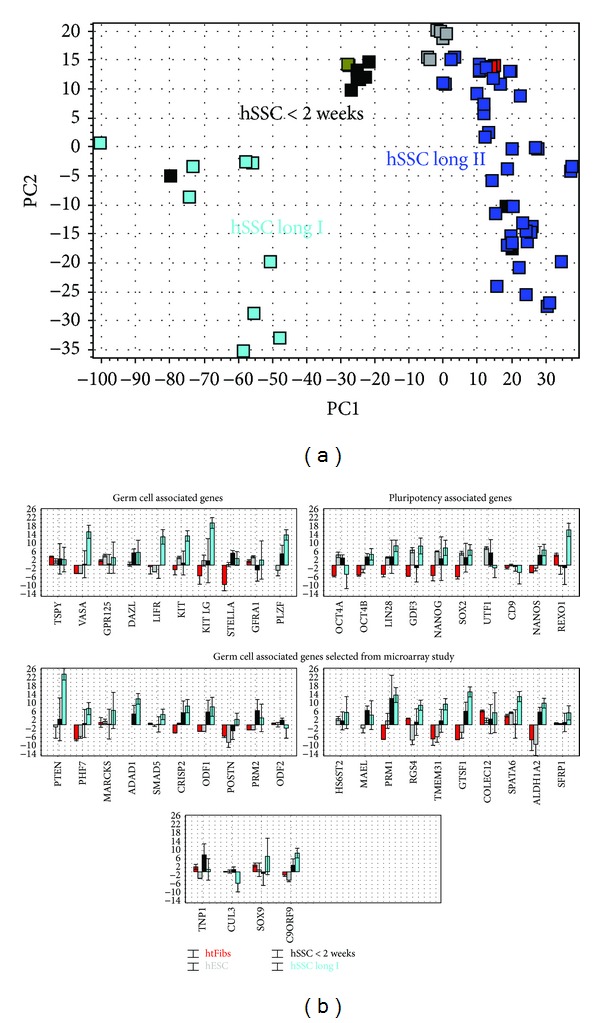
Analysis of all long-term cultured spermatogonia by real-time PCR. PCA shows that htFibs, hESCs, short-term cultured hSSCs, and a group of long-term cultured hSCCs (group I) clearly separate. (b) Bar plots of long-term cultured spermatogonia (group I) in comparison to short-term cultured spermatogonia, hESCs, and htFibs: (a) For germ cell-associated genes, (b) pluripotency-associated genes, and (c) germ cell-associated genes selected from microarray study. Coloring: htFibs: red; hESCs: grey; short-term cultured spermatogonia from patient 219: black; long-term cultured spermatogonia from group I: green; long-term cultured spermatogonia from group II: blue.

In an extended approach, we now considered different predefined sets of genes: fibroblast specific genes, hESC-enriched genes, and genes found to be enriched in fibroblasts. The genes were extracted from the publication of Ko et al. 2010 [22] (Figure 1k: human ES cell-enriched genes). Expression of the three different gene sets (fibroblast-specific genes, ES-enriched genes, and fibroblast-enriched genes) is presented in heatmaps in Figure S4 and in Figure 6(a) which corresponded to Figure 1k from the publication of Ko et al. [22].

Interestingly, gene* HOOK1* was strongly expressed in short-term cultured spermatogonia. In long-term cultured spermatogonia genes* LRRN1* and* CXADR*, both of which are linked to germ cells, were upregulated.

Short- and even more interestingly long-term cultured spermatogonia expressed several genes from the list of genes involved in extracellular matrix production and attachment (Figure S4). However, especially for the short-term cultured spermatogonia, there were substantial differences relative to htFibs.

A correlation matrix of the four different cell types based on the five gene sets (pluripotency genes, GS genes, fibroblast-specific genes, ES-enriched genes, and fibroblast-enriched genes) is shown in Figure S5. Short-term cultured spermatogonial cells showed similar correlation to hESCs (Pearson correlation coefficient *ρ* = 0.51) and to htFibs (*ρ* = 0.54). Short- and long-term cultured spermatogonia were more distinct (*ρ* = 0.43). In particular long-term cultured spermatogonia were different from hESCs based on this data subset (*ρ* = 0.08), but they were more similar to htFibs (*ρ* = 0.72) using five gene sets.

While htFibs were quite different from hESCs (*ρ* = 0.17), they had similarities to the short-term cultured spermatogonia (*ρ* = 0.54) and long-term cultured spermatogonia (*ρ* = 0.72). Since, according to morphological criteria, no fibroblasts were selected for the analysis from the germ cell cultures, the increase of fibroblast-enriched genes during long-term spermatogonia culturing may be related to culture conditions on feeder layers and growth factors.

#### 2.4.5. Differential Analysis of Microarray Data with Test of Germ Cell-Associated Terms

In a text mining approach we investigated if the germ cell-specific terms: “sperm,” “testis,” “meiosis,” “germ,” and “gamete” were annotated for the genes. To this end gene descriptions, retrieved from BioMart, were parsed for the occurrences of these terms. Features, whose description contain at least one of the terms, were coloured in a volcano plot of hSSC_short and control cells in Figure S5 and Table 1. An unbalanced left/right distribution of a colour indicates a relation of the annotated term with the sample/control grouping. For statistical assessment of this unbalance we tested whether log2 ratios for genes containing a certain term were identical to log2 ratios for genes that do not contain the term based on* t*-test and Wilcoxon's test. The results are shown in Table 1. Genes annotated with “sperm” or “testis” were significantly upregulated in hSSCs compared to the control group. For long-term spermatogonia this association was not observed.

The differential analysis of the microarray data clearly indicates that the CD49f and matrix selected short-term (<2 weeks) cultured cells were not bulk fibroblasts [18] but rather showed individual germ cell specific expression patterns. This broad variation in expression was due to cell type-specific genes and may serve as a fingerprint for spermatogonia.

### 2.5. Validation of Microarray Results by Nanofluidic Real-Time PCR

The initial set of markers for characterizing genes enriched in human SSCs included the germ cell-specific genes:* TSPYL*,* DDX4* (*VASA*),* DAZL*,* ZBTB16 *(*PLZF*),* DPPA3* (*STELLA*),* CD9*,* NANOS*,* UTF1, LIFR, KIT, KILG, REX1, GPR125, *and* GFRa1,* and the pluripotency-associated genes:* POU5F1* (*OCT4) A*,* LIN28A*,* NANOG*,* SOX2*, and* GDF3*. In addition the expression of OCT4B was measured. Based on the microarray data analysis of the group of short-term cultured spermatogonia, the following germ cell- and spermatogenesis-associated genes were selected for the real-time PCR analysis:* PRM1*,* PRM2 2*,* TNP1*,* CRISP2*,* ODF1*,* ODF2*,* ADAD1*,* PHF7*,* SPATA6*,* TMEM31*,* MAEL*,* C9ORF9,* and* GTSF1*, as based on high expression levels (see Tables S3A and S3C). For gene expression profiling of long-term cultured spermatogonia the following genes, associated with stem/germ cells and spermatogenesis, were selected:* PTN*,* MARCKS*,* SMAD5*,* POSTN*,* ALDH1A2*,* SRFP1*,* CUL3*,* HS6ST2*, SOX9,* COLEC12*,* TSPYL1,* and* RGS4* based on high expression levels (see Tables S3B and C).

#### 2.5.1. Human Testis Fibroblasts (htFibs)

According to the Fluidigm real-time PCR analysis 14 out of the 45 transcripts were found completely absent in somatic htFibs, including genes* DDX4* (*VASA*),* DAZL*,* UTF1*,* KITLG*,* ADAD1*,* CRISP2*,* ODF1*,* PRM1*,* PRM2*,* MAEL*,* GTSF1*,* PTN*,* HS6ST2,* and* GDF3*. Furthermore, the genes* STELLA* and* GFRA1 *showed very low expressions with CTs > 25 cycles in htFibs. These genes are all associated with germ cells but for GDF3, which belongs to the group of pluripotency enriched genes and may therefore be used as marker to distinguish human spermatogonia from testis fibroblasts.

#### 2.5.2. Short-Term Cultured Spermatogonia

When the expression of genes from the three subsets measured on the Fluidigm were compared between htFibs, hECss, and short-term cultured spermatogonia (patient 219) similar results to those from the microarray analysis (patients 189 and 195) were obtained. In hierarchical cluster analysis hESCs and htFibs localize to the same subtree and are clearly separated from the short-term cultured spermatogonia (Figure 6). Also from the PCA (Figure S6) it is evident that short-term cultured spermatogonia cells are different from the other cell types.

Several of the markers characteristic of germ cells:* DAZL, STELLA (DPPA3), PLZF (ZBTB16), NANOS, and UTF1, *and the pluripotency-associated genes* POU5F1A *and* SOX2* were enriched in the short-term cultured hSSCs (short-term spermatogonia <2 weeks, patient 219 versus htFibs, Mann-Whitney *P* < 0.05), while genes* TSPYL, VASA (DDX4), PLZF (ZBTB16), GPR125, LIN28, NANOG, GFR*α*-1, KIT LG, LIFR, *and* GDF3* were not significantly differentially expressed in this patient (short-term spermatogonia <2 weeks, patient 219 versus htFibs, Mann-Whitney *P* > 0.05). When two outliers were removed from the group of cells collected from patient 219, all genes but LIFR were now found significantly differentially expressed. The genes* PRM1*,* PRM2*,* TNP1*,* CRISP2*,* ODF1*,* ODF2, GTSF1,* and* MAEL* that were overexpressed in short-term cultured spermatogonia in the microarray analysis were also found overexpressed by real-time PCR, and we conclude that expression of these markers clearly distinguishes short-term cultured spermatogonia from hESCs and htFibs (Figure 6). The genes that had the highest expressions (fold change) in the short-term cultured spermatogonia in comparison to htFibs were* PRM1*, followed by* STELLA, GTSF1, ALDH1A2,* and* PLZF,* and in comparison to the hESCs they were* ALDH1A2, TNP1,* and* PRM1*. From the group of long-term enriched genes* GTSF1, ALDH1A1, MAEL, CRISP2, TMEM31,* and* ADAD1* were upregulated in short-term cultured spermatogonia compared to htFibs (Mann-Whitney test, *P* < 0.05) (Figure 6).

#### 2.5.3. Long-Term Cultured Spermatogonia and Overall Comparisons of Short- and Long-Term Cultured Spermatogonia

The PCA results in Figure 6 separating htFibs, human ES cells, short-term and long-term cultured spermatogonia were analysed in more detail. The following features were apparent: a compact cluster of “short-term cultured spermatogonia” samples from patient 219 with two outliners (red), a compact cluster of htFibs (purple), a compact cluster of hESCs (grey), and separation of “long-term cultured spermatogonia” into two major groups (green), a small group of nine samples (group I) from four patients, 184, 191, 201, and 203 (see Tables S1 and S2), and a large group of 40 samples (group II) from six patients, 184, 191, 196, 201, 203, and 214 (Figure 6).

The expression profile of group I consisted of the germ cell genes* VASA, PLZF, LIFR, KITLG, KIT STELLA, DAZL, REXO1, *and* TSPY1* and the pluripotency-associated genes* NANOS, LIN28, NANOG, SOX2, *and* REXO1, *from thedifferentiation pathway* CRISP2, ODF1, and PRM1,* and from the group of long-term cultured associated genes PHF7, POSTN, TMEM31, PTEN, ADAD1, ALDH1A2, SMAD5, RGS4, SPATA6, SFRP1, CUL3, and* C9ORF9 *(Mann-Whitney test, *P* < 0.05, compared to htFibs) (Figure 6). In group II only the genes* OCT4A, OCT4B, PHF7,* and* POSTN* were significantly upregulated relative to htFibs (Mann-Whitney test, *P* < 0.05, compared to htFibs).

In general, the “long-term cultured spermatogonia” samples had similar PC1 scores, suggesting that expression of the genes behind PC1 is fairly homogeneous among those samples, while the genes behind PC2 show variability (Figure S6). Interestingly, the PC1 score for the “long-term cultured spermatogonia” samples is similar to that of the htFibs and of the hESCs, suggesting that they have common expression of the underlying genes. In this analysis all plots reveal four “long-term cultured spermatogonia” and two “short-term cultured spermatogonia” outlier samples characterized by a very low PC1 score. When including more genes in the classification the separation of htFibs and “long-term cultured spermatogonia” disappears in the PC1 versus PC2 plot. However, it remains in the 3D plot that reflects also the contribution from PC3, evidencing different expression profiles (Figure S6).

Notable, the “short-term cultured spermatogonia” samples are in the centre, while the “long-term cultured spermatogonia” samples are located at the periphery of the PC1 versus PC2 plot. The separation is even more evident in a 3D PC1 versus PC2 versus PC3 plot. This evidences samples changing expression profiles as the spermatogonia ages in the culture, and the changes are not homogeneous. Rather, the samples differentiate into three different directions reflected by moving towards high PC1 or high or low PC2. The genes important for the separation along the three axes include* ALDH1A2*,* PHF7*,* NANOG*,* LIN28*,* SOX2*,* MARKS,* and* POSTN*.

The expression profiles of germ cell-enriched genes in short- and long-term cultured spermatogonia in comparison to htFibs were heterogeneous across the different patients, with minor differences (Figure S7). The observed variation in expression profiles might be related to the heterogeneous pathologies of the patients (Table S1). Contributions from individual variation were not explicitly studied here. Previous works have shown that individual variation is gene dependent, but generally much lower than the differential expression observed here for the important markers [23]. Individual variation is also expected to be mitigated by culturing of the cells. In cases when cells from several individuals were cultured in replicates under the same conditions and length we find a tendency for the replicates for subgroups in multivariate analysis, indicating individual variation is larger than that of technical replicates, which is expected. But the main clusters separate differentially cultured cells and different cell types, evidencing that this is the dominant effect to their distinct expression profiles.

Among the studied germ cell-associated genes* STELLA, CD9*, PLZF, and DAZL were upregulated relative to htFibs in all cultures of spermatogonia across several different patients in comparison to htFibs (Mann-Whitney test, *P* < 0.05, compared to htFibs). The gene* POSTN* was the most homogenously upregulated gene in this group. Furthermore among the pluripotency genes* POU5F1A*,* NANOG, *and SOX2 were expressed to higher level and also* POSTN, TMEM31, ALDHA2, OCT4b, NANOS, C9ORF9, GTSF1, *and* GDF3* (Mann-Whitney test, *P* < 0.05, compared to htFibs, Figure S6).

## 3. Discussion

The results presented in this study show that short-term cultured human spermatogonia (<2 weeks) can be highly enriched by CD49f MACS and matrix selection and furthermore long-term cultured. Because MACS/matrix enrichment generates a heterogeneous population of cells dominated by spermatogonia, cells with typical spermatogonial morphology were collected with a micromanipulator for microarray and real-time PCR analyses to measure gene expression profiles of adult human spermatogonia during short- and long-term culture.

We used a complex culture system with growth factors GDNF, LIF, bFGF, and EGF that supported the CD49f/matrix selected spermatogonia increasing their number by self-renewal. Using this protocol spermatogonial cells could be maintained* in vitro* for more than a year [5]. The proliferation of the cells in culture was slow. In short- and long-term cultures single as well as aligned human spermatogonia were observed. Furthermore, small grape-like aggregates of spermatogonia and also colonies of spermatogonia were present in the cultures. The aligned cells formed pairs or chains of four to eight cells, typically for more differentiated spermatogonia. In addition to the spermatogonia also some more differentiated large round cells were also present, which eventually died. This observation confirmed that MACS/matrix selection provided a heterogeneous population of cells with predominating spermatogonia and that differentiation occurred during culture. Recently, also other groups have established successful short- and long-term cultures of spermatogonia from humans with rather high proliferative capabilities [3, 6, 8, 11].

Our results show that many well-known germ cell-associated genes and pluripotency-associated genes are expressed by short-term cultured spermatogonia and also in a minor population of long-term cultured spermatogonia. The genes include* DDX4* (*VASA*) [24],* DAZL *[25–27], and* ZBTB16 *(*PLZF*) [28, 29].

In addition to the established spermatogonial markers, short-term cultured spermatogonia were found to express the ES cell-associated gene* HOOK1* in this study.* HOOK1* is a cytosolic coiled-coil protein attached to microtubules that mediates binding to cell organelles. It is found at high levels in human testes [30]. The loss of* HOOK1* gene function results in ectopic positioning of microtubular structures within the spermatids [31]. Additionally, several genes important for spermatogenesis, including* PRM1*, PRM*2, ODF1,* and ODF*2,* were found expressed in short-term cultured and selected spermatogonia in this study.

The majority of long-term cultured spermatogonia lost the expression of the germ cell-specific genes* VASA*,* DAZL*, and* PLZF*, but still expressed other important germ cell-associated genes including* STELLA* (*DPPA3*) and* LIN28*. Bowles et al. [32] identified the gene* DPPA3*,* developmental pluripotency-associated gene 3*, which encodes a 160-amino acid protein. In humans* DPPA3* is expressed mainly in germ cell tumors, but not in normal testes. It has been shown that mouse embryonic stem cells carrying a STELLA transgenic reporter can be differentiated into putative primordial germ cells (PGCs)* in vitro* [33]. It is interesting that* LIN28 *and* STELLA* somehow interact. Overexpression of* LIN28* promotes the formation of STELLA-positive cells* in vitro* and of PGCs in chimeric embryos, and it is associated with human germ cell tumors. Perhaps the expression of these genes by the long-term cultured spermatogonia in this study reflects a more the more undifferentiated germ cell like profile.

In addition, long-term cultured spermatogonia expressed* PTN*,* SFRP1*,* POSTN*,* PTEN*,* MARCKS*,* SMAD5*,* CAR*,* ALDH2*,* CUL3*,* TSPYL1,* and TXNIP.* PTN*, pleiotrophin, is a heparin binding secretory growth/differentiation factor. The study by Zhang et al. [34] supported a central role of* PTN *signaling in normal spermatogenesis and suggest that interruption of* PTN* signaling may lead to sterility in mice.

The high expression of* SFRP1*, frizzled related protein 1, which was observed in the long-term cultured spermatogonia may be explained by their similarity to primordial germ cells, which undergo global demethylation [35]. SFRP1 protein is a soluble Wnt antagonist, which has been suggested as tumor suppressor [36]. The inhibition of* WNT* transcription and activation of* NOTCH* are features of undifferentiated human embryonal carcinoma and ES cells [37], and may be mediated by the* SFRP1 *gene, which is repressed in differentiating NT2/D1 cells (derived from human EC cells) after treatment with retinoic acid [38]. High expression of SFRP1 is also observed in testis carcinoma* in situ* cells. The long-term cultured spermatogonia studied here also expressed periostin (*POSTN), which* is a member of the fasciclin family and a disulfide-linked cell adhesion protein. POSTN product interacts with multiple cell-surface receptors, most notably integrins, and signals mainly via the PI3-K/Akt pathway [2]. It has been shown that POSTN is involved in the development of various tumors [39].

The gene* CAR*, coxsackie and adenovirus receptor, functions as an adhesion molecule between Sertoli and germ cells at the Sertoli-germ cell interface [40]. Immunofluorescence staining of isolated testicular germ cells has revealed expression of CAR on spermatogonia, spermatocytes, round spermatids, and elongate spermatids. Similarly, Mosevitsky et al. [41] found that MARCKS appeared to be present at similar level during all stages of spermatogenesis, except in mature spermatozoa.

Long-term cultured spermatogonia in this study also expressed* SMAD5*. It has been shown that expression of* SMAD5* is required for PGC development in mice [42].* SMAD5* gene expression in the testis has been localized to stem and differentiating spermatogonia and has provided insights into the BMP regulation of spermatogenesis [43].* SMAD5* may interact with* BMP4* and have a role in spermatogonia differentiation [44].

Also other genes found expressed by long-term cultured spermatogonia have already been confirmed during testis development and spermatogenesis. Wu et al. [45] found expression of* ALDH2*, retinoic acid-metabolizing enzymes, during mouse postnatal testis development.* CULLIN3* is a KLHL10-interacting protein preferentially expressed during late spermatogenesis [46].* TSPYL1* is expressed by spermatogonia and mutations have been associated with disorder of male sex development and infertility [47].* Thioredoxin-interacting protein (TXNIP)* has been associated with the formation of germ cells from embryonic stem cells in cultures with high glucose concentrations [48]. It has also been found highly expressed in adult and embryonic gonads.

Long-term cultured spermatogonia were here shown to be a heterogeneous population of cells, although still retaining the expression of several genes of pluripotency (especially* LIN28, SOX2, *and* NANOG*). This may reflect the* in vitro* culture condition and may be related to cell de-differentiation towards the more undifferentiated germ cell-like profile. A population of the long-term cultured cells retained and even aggravated the spermatogonial profile with the expression of germ- and pluripotency-associated genes.

Our molecular analyses of short- and long-term cultured human spermatogonia supports previous research on the culturing and propagation of human spermatogonial stem cells [9] but also raises several important issues in terms of their changes during* in vitro* culture.

While the short-term cultured spermatogonia shows the full spectrum of stem- and germ cell/differentiation-associated genes, the majority of long-term cultured spermatogonia had lost the expression of several important genes related to germ cell differentiation (e.g.,* VASA*,* DAZL*, and* PLZF*) and obtained more “basal” gene expression profile. This profile may play a role in promoting the long-term proliferation of spermatogonia* in vitro*. Differential analyses of microarray expression data in this study clearly indicate that the CD49f and matrix selected short-term cultured spermatogonia were not merely fibroblasts [22] but rather showed individual germ specific expression patterns. This broad pattern was composed of germ cell type-specific and pluripotency-associated genes and may serve as transcriptional fingerprint for spermatogonia. It also became evident from the molecular analysis that a minority of cultured cells maintained a full expression spectrum of germ cell specific genes, including genes of pluripotency. This observation was possible by analysis of small samples (50 cells) with nanofluid real-time PCR technology and would have been overlooked by the inherent averaging of individual profiles when studying conventional, much larger samples. These observations indicate that the selection criteria for spermatogonia still have to be improved. Other cell surface markers in addition to the CD49f/matrix possibly combined with manual selection have to be experimentally tested to further enrich stem cell spermatogonia during culturing. It also became evident from our study that the quality of the cultures is largely dependent on the starting material, that is, the patient's testis tissue with the pathological background. In this study only pathological tissue with limited quality was available for the experiments.

At present it is unclear if the cultured spermatogonial cells retain their full differentiation capacity of spermatogenesis after transplantation. The approach we provide to study the molecular status of* in vitro* cultured spermatogonia may be important to optimize the culture conditions and to evaluate cell plasticity in future studies. The differentiation models for spermatogenesis must be improved to unequivocally demonstrate the functional properties of the cultured human spermatogonial cells based on some new findings, such as the* model* of* in vitro* differentiation of long-term cultured adult human spermatogonia [9] or the organotypic slice culture system for* in vitro* spermatogenesis in mice [49]. Further studies of long-term cultured human spermatogonia are required to improve their proliferative capacities* in vitro. *This should also be accompanied by a more detailed genetic, epigenetic, and functional characterisation of* in vitro* cultured human SSCs, which have potential to become of major clinical importance.

## 4. Materials and Methods

### 4.1. Patients and Testicular Tissue

The study was performed from October 2009 until June 2011 using testicular tissue from 10 adult men with different medical backgrounds. The experiments with human material were approved by the Ethical Committee of the Medical Faculty and University of Tübingen, Chairman Professor Dr. Luft, reference number 493/2008A and Ethical Committee of the Medical Faculty of the University of Heidelberg, Chairman Professor Dr. Strowitzki, reference number S-376/2010. Informed written consent was obtained from all the human subjects. The age of the patients ranged from 20 to 84 years. Healthy, nonmalignant donated testicular tissue included heterogeneous material from patients with different medical backgrounds, including orchiectomies as part of prostate cancer treatment (1 patient), reassignment surgeries of transsexual patients after hormone therapy (6 patients), diagnostic testicular biopsy (1 patient), and a healthy testicular tissue biopsy from 1 patient with seminoma (Table S1). Histopathological examinations of the tissue used in this study were performed at the Department of Pathology (University of Tübingen) in routine diagnostics and diagnoses are presented in Table S1.

### 4.2. Experimental Design

In this study gene expression profiles of short-term (<2 weeks) and long-term (up to 14 months) cultured spermatogonia of ten men were analyzed. In three men (patients 184, 194, and 195) spermatogonia after one short- and three long-term cultures were analyzed based on gene expression profiles using the BioMark (Fluidigm) for a pilot study (Table S2). In three men (patients 184, 189, and 195) spermatogonia after two short-term cultures and one long-term culture were analyzed by microarray profiling (Table S2). In seven men, including one short-term cell culture (patient 219) and six long-term cell cultures (patients 184, 191, 196, 201, 203, and 214) the microarray data were validated by real-time PCR using the BioMark (Fluidigm) system (Table S2). Spermatogonial gene expression profiles were compared to those of hESCs and htFibs.

### 4.3. Selection and Cultivation of hSSCs

After removing of the tunica albuginea, the obtained human testis tissues were mechanically disrupted to dissociate the tubules. The dissociated tubules were transferred for enzymatic digestion into 750 U/mL collagenase type XI (Sigma), 0.25 *μ*g/mL dispase II (Roche), and 5 *μ*g/mL DNase in HBSS buffer with Ca^++^ and Mg^++^ (PAA) for 30 minutes at 37°C, with gently mixing, to obtain a single cell suspension. Then the digestion was stopped with 10% ES cell qualified FBS, given through a 100 *μ*m cell strainer and centrifuged at 1000 rpm for 15 minutes. The supernatant was removed and the pellets were washed with HBSS buffer with Ca^++^ and Mg^++^. After washing, the cells were plated into 10 cm culture dishes, coated with 0,2% gelatin (Sigma) and 2 × 10^5^ cells per cm^2^, containing hGSC (human germ stem cell) medium with StemPro hESC medium (Invitrogen), 1% N2-supplement (Invitrogen), 6 mg/mL D+ glucose (Sigma), 5 *μ*g/mL bovine serum albumin (Sigma), 1% L-glutamine (PAA), 0,1% *β*-mercaptoethanol (Invitrogen), 1% penicillin/streptomycin (PAA), 1% MEM vitamins (PAA), 1% nonessential amino acids (PAA), 30 ng/mL estradiol (Sigma), 60 ng/mL progesterone (Sigma), 20 ng/mL epidermal growth factor (Sigma), 10 ng/mL basic fibroblast growth factor (Sigma), 8 ng/mL glial-derived neurotrophic factor (GDNF; Sigma), 100 U/mL human LIF (Millipore), 1% ES cell qualified FBS, 100 *μ*g/mL ascorbic acid (Sigma), 30 *μ*g/mL pyruvic acid (Sigma), and 1 *μ*L/mL DL-lactic acid (Sigma), and incubated at 37°C, 5% CO_2_ for 96 hours. At day 7 the culture medium was removed and the testis cell cultures were gently rinsed with 5 mL of DMEM/F12 with L-glutamine (PAA) per plate to harvest the bound germ cells from the monolayer of adherent somatic cells by repeated pipetting with 5 mL of DMEM/F12 medium. The pooled suspension retrieved from 5 culture dishes was centrifuged at 1000 rpm for 5 minutes, suspended in 10 mL hGSC medium and plated onto one plate with collagen type I-coated 10 cm culture dish and incubated for 4 hours in the CO_2_-incubator. After the incubation the cells were rinsed again with the used medium, collected in 15 mL Falcon Tube, and centrifuged for 10 minutes at 1000 rpm. The pellet was resuspended in 10 mL of MACS buffer and centrifuged again for 5 minutes and the cells were further purified with MACS separation (Miltenyi), CD49f-FITC (*α*_6_-integrin; AbD serotec), and anti-FITC beads (Miltenyi). After MACS separation, positive cells from original five 10 cm dishes were plated onto 1 well of a 12-well plate on irradiated CF-1 feeder in hGSC media. Half of the media were removed every 2-3 days and replaced with half of fresh hGSC media. Under these conditions the spermatogonia proliferated quiet heterogeneously. The cultures were split (1 : 2) every two to three weeks. It was important not to stronglysplit the cellsand to keep all the time the appropriate cell number in the wells. hESCs and htFibs were collected in the same way.

### 4.4. Collection of Spermatogonia with a Micromanipulation System

To collect the spermatogonia for different experiments, 50 or 200 spermatogonial cells per sample/patient were collected in the first two weeks for original short-term culture or after 1, 4, 6, 8, 11, and 14 months for long-term culture experiments (see Figure S2). The cells were rinsed with the used culture media to remove the spermatogonia from the monolayer of somatic cells or feeder layer. The cells were transferred after gentle resuspension to a single cell suspension into the top of small culture dishes (*d* = 3.5 cm). The tops of the dishes were placed onto a prewarmed (37°C) working platform of an Axiovert 200 inverted microscope (Zeiss) with hydraulic micromanipulator (Narishige). At magnification 20x, the single spermatogonia were collected step by step under the criteria of typical spermatogonia morphology (only small round cells with a diameter of 6–12 *μ*m and high nucleus/cytoplasm ratio observed by round yellow shining nucleus and a small white cytoplasmic ring). Each spermatogonium was aspirated into the glass micropipette and transferred under visual control into the droplet of culture medium. The transfer of each spermatogonium was monitored both in the pipette and in the droplet. Fifty cells per sample probe were collected for Fluidigm analysis and 200 cells per probe for microarray analysis. After collection, the cells were transferred directly into 6.5 *μ*L of CellsDirect buffer (Invitrogen) for Fluidigm or 10 *μ*L RNA direct lyses buffer for microarray analysis.

### 4.5. Microarray Gene Expression Analysis

Total RNA isolated for short-term culture (during the first two weeks after matrix selection) was obtained from two independent cell cultures of spermatogonial cells from two patients (Table S1: patients 189 and 195), for long-term (up to 14 months) cultured spermatogonia from three independent cell cultures from one patient (Table S2: patient 184), from htFibs, and the hESC line H1 (WA01, WiCell) from 3 independent cell cultures and prepared using the RNeasy Mini Kit (Qiagen) followed by an amplification step with MessageAmp aRNA Kit (Ambion). Two hundred cells were collected per probe with a micromanipulator and transferred directly into 10 *μ*L RNA direct lysis solution and stored at −80°C. Samples were provided for analysis to the Microarray facility of University Clinic, Tübingen. Gene expression analysis was performed using the Human U133 + 2.0 Genome oligonucleotide array (Affymetrix). The raw data (CEL-files) was provided to MicroDiscovery GmbH, Berlin, Germany, for normalization and biostatistical analysis. Differential analyses were used to define lists of known germ cell-associated genes upregulated in short (SSC_short:* PROTAMIN 1*,* PROTAMIN 2*,* TNP1*,* CRISP2*,* ODF1*,* ODF2*,* ADAD1*,* PHF7*,* SPATA6*,* TMEM31*,* GSF1*,* MAEL*,* C9ORF9*, and* GTSF1*) and long-term cultured spermatogonia (SSC_long:* PTN*,* MARCKS*,* SMAD5*,* POSTN*,* ALDH1A2*,* SRFP1*,* CUL3*,* HS6ST2*,* PTEN*,* LIFR*,* CCND2*,* CXADR*, and* RGS4*) when compared to testis htFibs and hESCs. Additional sets of literature-derived pluripotency-associated genes (Pluripotency:* POU5F1 (OCT4)*,* LEFTY2*,* CDX2*,* FOXO1*,* LIN28*,* HAND1*,* DPPA4*,* SOX2*,* ZIC3*,* GJA1*, and* NANOG*) and germ cell-enriched genes present in germ cells that are not specific for spermatogonia (GS genes:* TSPY1*,* DDX4* (*VASA*),* UTF1*,* CD9*,* ZBTB16* (*PLZF*),* TSPYL1*,* GPR125*,* DAZL*,* GFR*α*-1*,* DPPA3* (*STELLA*),* NANOS2*,* NANOS1*,* NANOS3*,* KIT*, and* LIFR*) were analyzed. In an extended approach we considered different predefined sets of genes from the literature: fibroblast-specific genes, hESC-enriched genes, and a list of genes found to be enriched in fibroblasts. All lists were extracted from the publication of Ko et al. 2010 (Figure 1k: human ES cell-enriched genes). Additionally, differential analysis of microarray data was performed by testing of germ cell-associated terms “sperm,” “testis,” “meiosis,” “germ,” and “gamete.” We investigated if these terms were annotated for the genes. To this end gene descriptions, retrieved from BioMart, were parsed for occurrences of these terms.

### 4.6. Microarray Data Normalization

Microarray data was imported into R Statistical Environment version 2.12.1 (2010-12-16). Data condensation was performed using Bioconductor package affy version 1.28.0. The condensation criteria were bg.correct = FALSE, normalize = FALSE, pmcorrect.method = “pmonly,” summary.method = “medianpolish.” Additional normalization was performed between samples using multi-lowess algorithm, a multidimensional extension of lowess normalization strategy [50]. Additional data are presented in a Supplemental Methods section.

### 4.7. Statistics of Microarray Data

The heatmaps and dendrograms of sample relations were used to evaluate the distances or degree of relationship between the compared experimental samples: SSCs, hESCs, and htFbs by calculating the whole assessed transcriptome, features with highest variance, or features with highest discrimination power. All heatmaps and dendrograms were created using hierarchical complete linkage clustering with Euclidean distance. The volcano plots were used to visualize fold changes and* t*-test *P* values of the comparisons of short-term and long-term cultured spermatogonia with the control groups htFibs and hESCs. The statistical significance of gene expression differences between different groups of cells (hSSCs, hESCs, and htFbs) was evaluated by different statistical tests:* F*-test,* t*-test, Pearson correlation coefficient, Wilcoxon test, and Mann-Whitney test. Significance criterion was *P* < 0.05.

### 4.8. Gene Expression Analyses on the Fluidigm BioMark System

To characterize the spermatogonial cells cultured under different conditions we first used the dynamic array chips (Fluidigm) to measure the expression of multiple genes in a very small number of cells. In samples from three men (Table 2 S2) spermatogonia after one short- and three long-term cultures were analyzed. In seven men (one short-term cell culture and six long-term cell cultures) microarray data were validated on the BioMark (Fluidigm) system (Table S2).

For each sample 50 spermatogonia, 50 control htFibs or 50 hESCs were manually selected from the different cell cultures with a micropipette and a micromanipulator. The pooled cells from each group were lysed, mRNA was reverse-transcribed into cDNA, which was sequence-specifically preamplified in a single tube, and the amount of targeted transcripts was quantified using TaqMan real-time PCR on the BioMark system (Fluidigm). Gene expression cell analysis of spermatogonia (50 cells per sample) in comparison with human embryonic stem cells (positive control, 50 cells per sample) and htFibs (negative control, 50 cells per sample) was performed using the BioMark Real-Time quantitative PCR (qPCR) system (Fluidigm). In all cell samples expression of up to 63 genes was measured: germ cell-specific genes (expressed in human and mouse spermatogonia):* TSPYL*,* DDX4 *(*VASA)*,* DAZL, ZBTB16 (PLZF), DPPA3 (STELLA), CD9, NANOS, UTF1 GFR*α*-1, KIT, KITLIG, DNMT3B, GPR125, *and* LIFR*; pluripotency-associated genes:* POU5F1 (OCT4A), POU5F1 (OCT4B), LIN28, NANOG, SOX2, GDF3, LIN28B, CDH1, TDGF1, TERT, MYC, STAT3, KLF4, *and* REX1*; germ cell-enriched genes (known as germ cell genes but normally are not used to characterize spermatogonia):* PROTAMIN1 (PRM1), PROTAMIN 2 (PRM2), TNP1, CRISP2, ODF1, ODF2, ADAD1*,* PHF7*,* SPATA6*,* TMEM31*,* GSF1*,* MAEL*,* C9ORF9*,* GTSF1*,* PTN*,* MARCKS*,* SMAD5*,* POSTN*,* ALDH1A2*,* SRFP1*,* CUL3*,* HS6ST2*,* COLEC12*,* C9ORF9*,* TSPYL1*,* RGS4, PTEN, *and* SOX9; *fibroblast-associated genes:* COL1A1*,* COL1A2*,* FIBRONECTIN1, *and* VIMENTIN*; and the housekeeping genes* CTNNB1*,* HNBS,* and* GAPDH*.

For preamplification the inventoried TaqMan assays (63x, Applied Biosystem) were pooled to a final concentration of 0.2x for each of the 63 assays. Cells to be analysed were harvested directly into 9 *μ*L RT-PreAmp Master Mix (5.0 *μ*L CellsDirect 2x Reaction Mix (Invitrogen); 2.5 *μ*L 0.2x assay pool; 0.2 *μ*L RT/Taq Superscript III [Invitrogen]; 1.3 *μ*L TE buffer). The harvested cells were immediately frozen and stored at −80°C. Cell lysis and sequence-specific reverse transcription were performed at 50°C for 15 min. The reverse transcriptase was inactivated by heating to 95°C for 2 min. Subsequently, in the same tube, cDNA went through limited sequence-specific amplification by denaturing at 95°C for 15 s and annealing and amplification at 60°C for 4 min for 14 cycles. These preamplified products were diluted 5-fold prior to analysis with Universal PCR Master Mix and inventoried TaqMan gene expression assays (ABI) in 96.96 dynamic arrays on a BioMark system. Each sample was analyzed in three to four technical replicates. *C*_*t*_ values obtained from the BioMark system were transferred to the GenEx software (MultiD) for analysis. Additional information on GenEx analysis is presented in a Supplemental Methods section.

### 4.9. Immunocytochemistry

To characterize human spermatogonia, we examined the expression of a panel of cell-specific proteins including CD49f, Dazl, VASA, STELLA, and the somatic marker VIMENTIN.

*Antibodies and Staining*. The following primary antibodies were used: mouse monoclonal biotinylated anti-CD49f (BioLegend), mouse monoclonal anti-Dazl (AbD Serotec), goat polyclonal anti-VASA (R&D Systems), rat monoclonal anti-STELLA (R&D Systems), and mouse monoclonal anti-VIMENTIN (Dako). The following secondary antibodies were used: Cy3 conjugated Streptavidin (Dako), Alexa Fluor-488 conjugated goat anti-mouse IgG (Molecular Probes), Cy3 conjugated rabbit anti-goat IgG (Dianova), and Cy3 conjugated goat anti-rat IgG (Dianova). All staining were costained with DAPI.

## Supplementary Material

In the supplements 7 Figures including detailed bar plots of Fluidigm real-time PCRs with germ, pluripotency and fibroblast-related gene expression profiling of htFibs, hES, hSSC are shown, followed by more volcano-blots and heat maps displaying various aspects of microarray analysis and real-time PCRs validating the microarray experiments. Furthermore Supplements Tables with patient's data, experimental design and the most up-regulated genes in the different comparisons between htFibs, hESC and hSSC according to the microarray experiments are provided. In the Supplements methods section more details about data normalization for the microarray analysis and GenEX analysis for Fluidigm real-time PCR data are provided.

## Figures and Tables

**Table 1:** Short-term cultured spermatogonia were significantly represented in a statistical evaluation of this unbalance.

WordStat-sample-hSSC_short versus control-hESC and -htFib and -hSSC_long				
	Number	Mean difference	*t*-test	Wilcoxon *P* value
Sperm	162	0.6219	3.334*e* − 10	1.208*e* − 15
Testis	72	0.9562	6.729*e* − 10	4.69*e* − 19
Meiosis	10	0.3853	0.04165	0.01713
Germ	8	0.4991	0.1311	0.02802
Gamet	7	1.774	0.03857	0.001336
