# Supplementary material for: Differential Gene Expression Profiling of Enriched Human Spermatogonia after Short- and Long-Term Culture
Source: Biomed Res Int. 2014 Mar 12;2014:138350. doi: 10.1155/2014/138350 (PMC3971551; doi:10.1155/2014/138350)
Supplement: Supplementary file 1 — In the supplements 7 Figures including detailed bar plots of Fluidigm real-time PCRs with germ, pluripotency and fibroblast-related gene expression profiling of htFibs, hES, hSSC are shown, followed by more volcano-blots and heat maps displaying various aspects of microarray analysis and real-time PCRs validating the microarray experiments. Furthermore Supplements Tables with patient's data, experimental design and the most up-regulated genes in the different comparisons between htFibs, hESC and hSSC according to the microarray experiments are provided. In the Supplements methods section more details about data normalization for the microarray analysis and GenEX analysis for Fluidigm real-time PCR data are provided. [file 138350.f1.doc]

**Supplementary Figure 1**


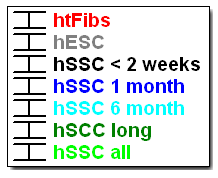
**A**


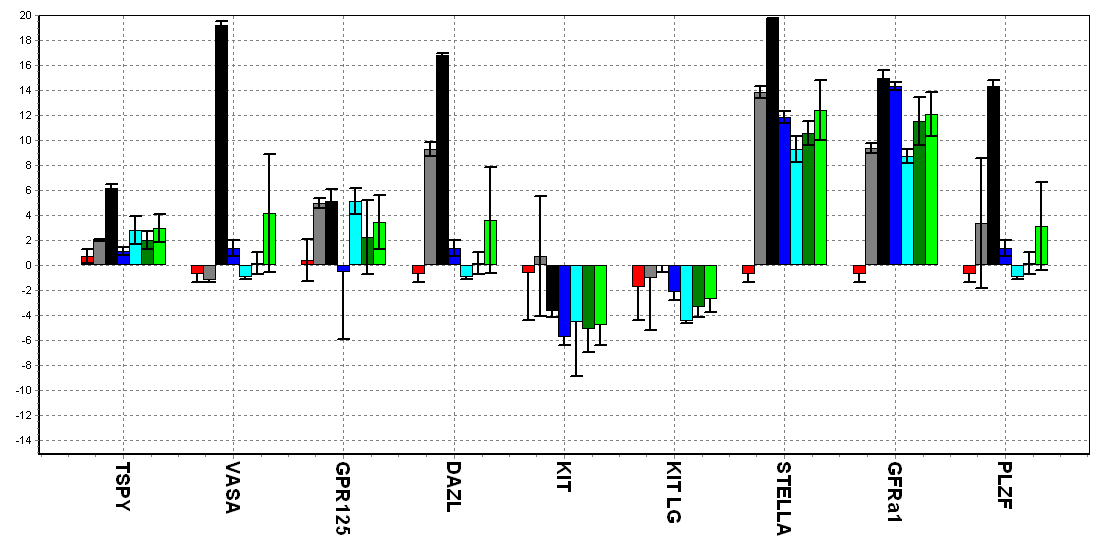

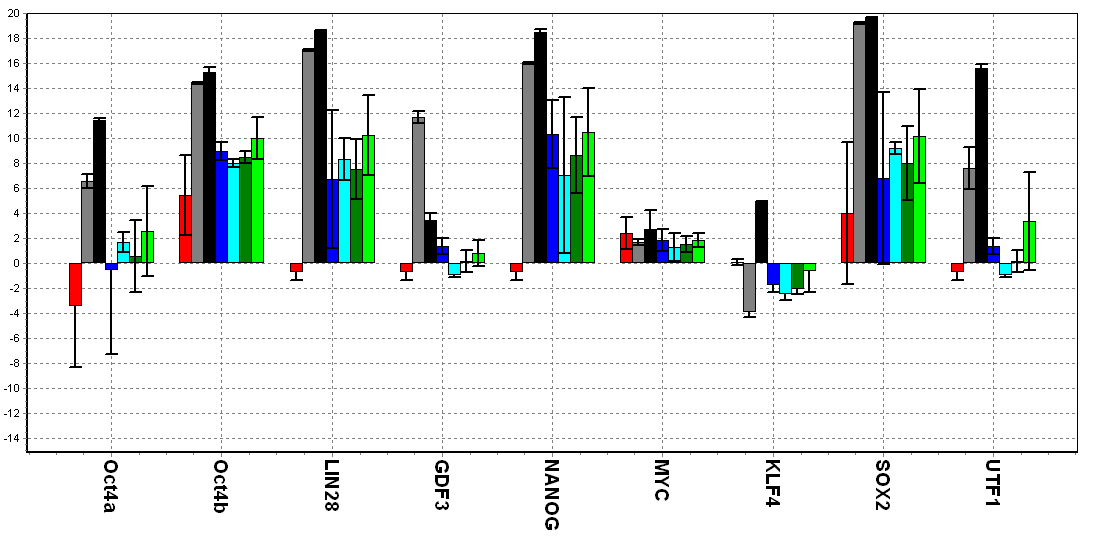
**germ cell, pluripotency and fibroblast associated genes**

**
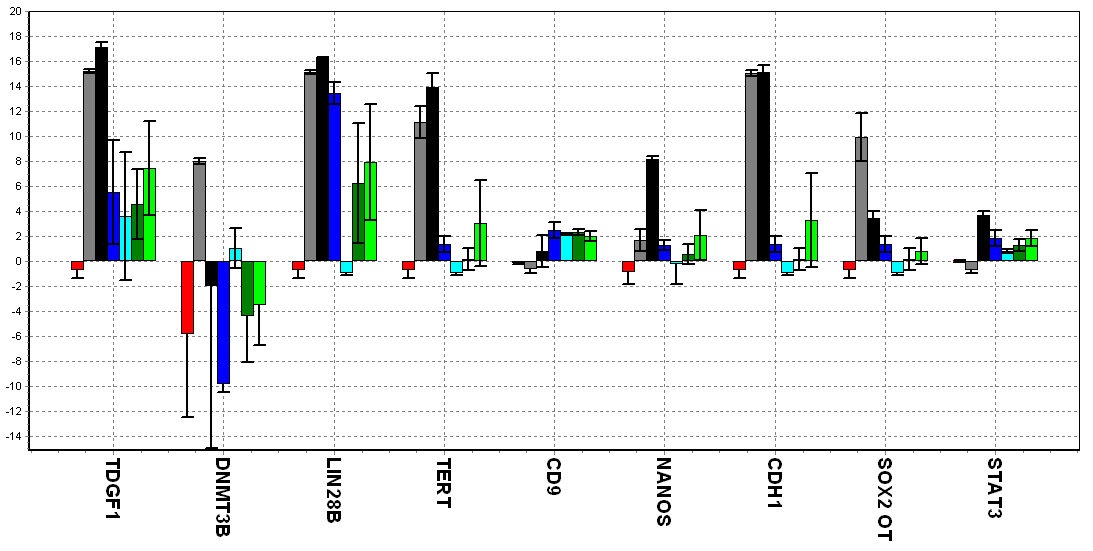

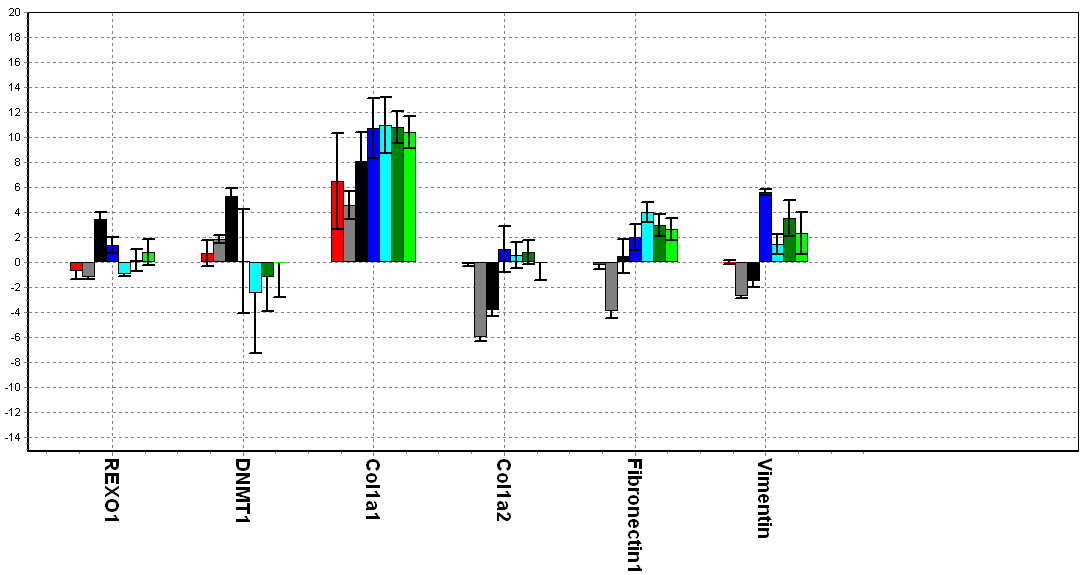
**


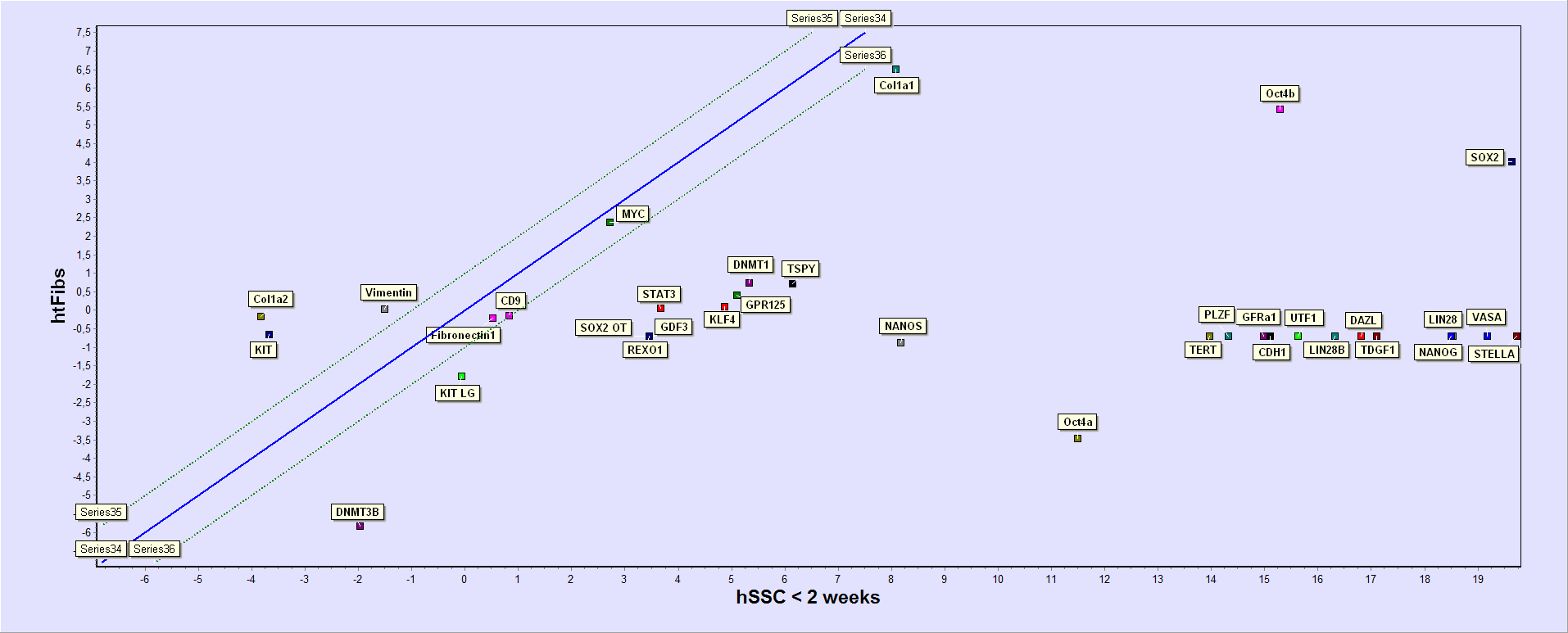

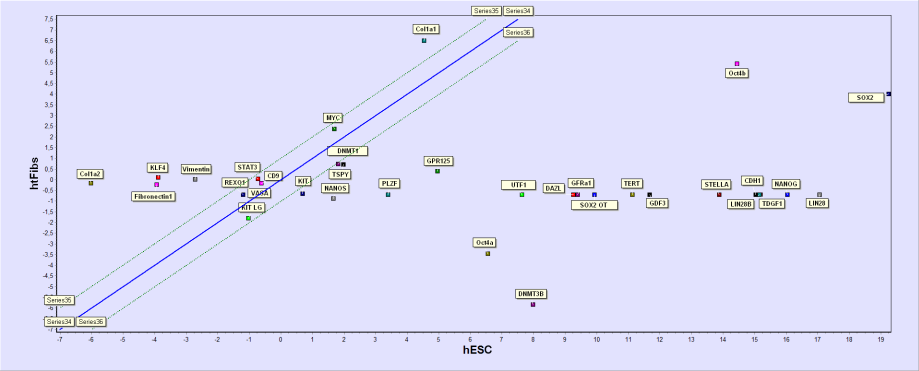
**B**

**
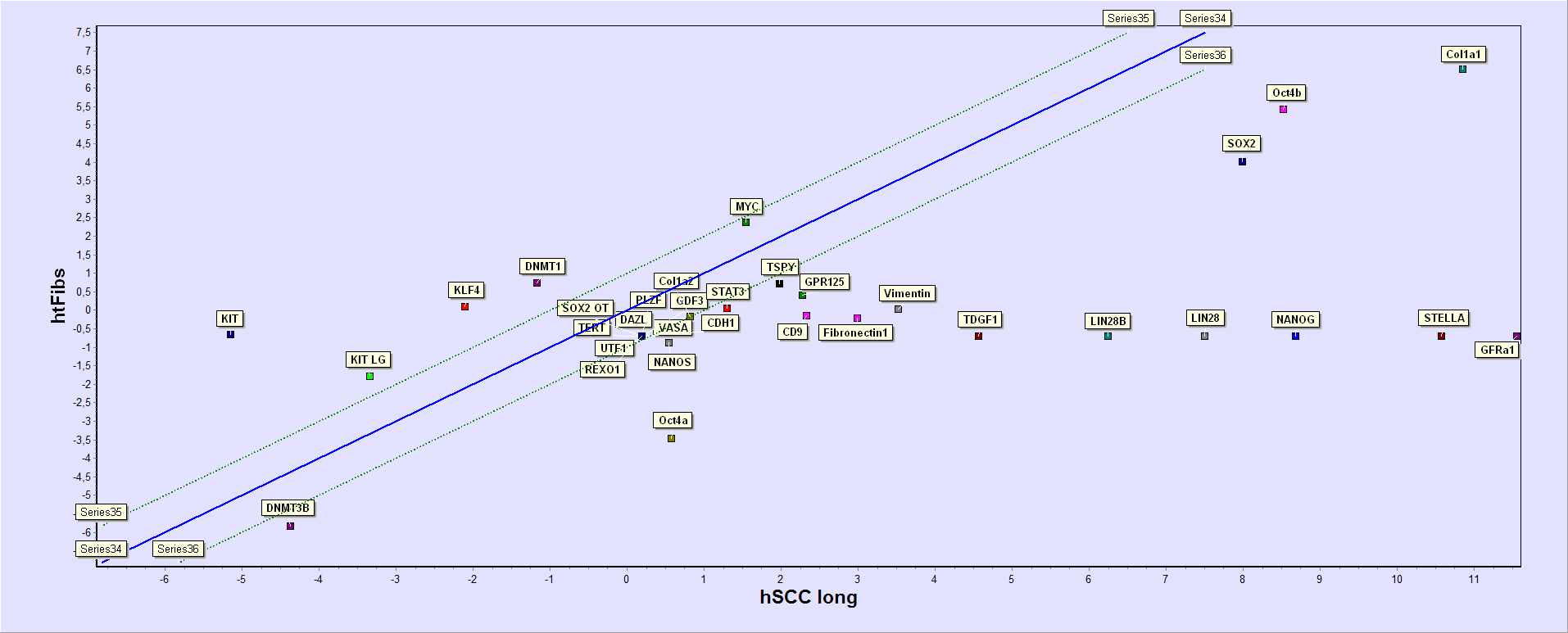
**

**Supplementary Figure 2**


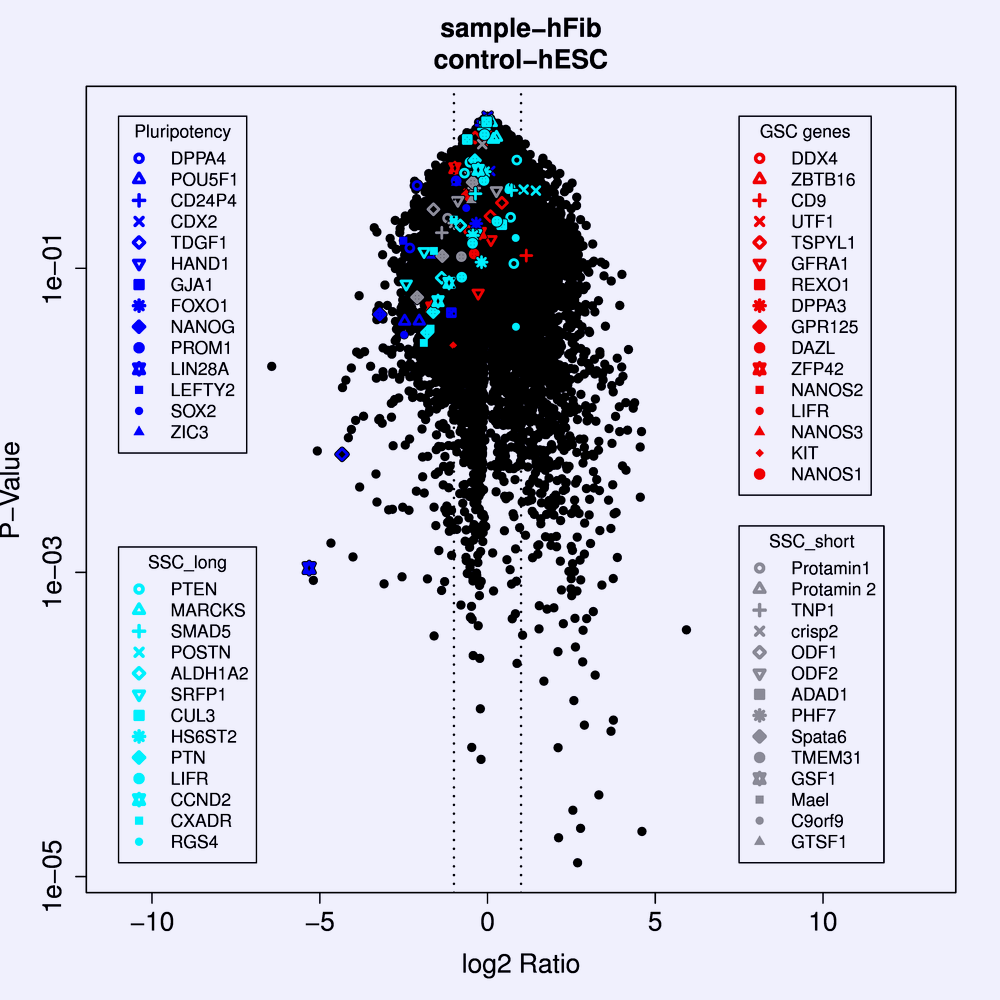

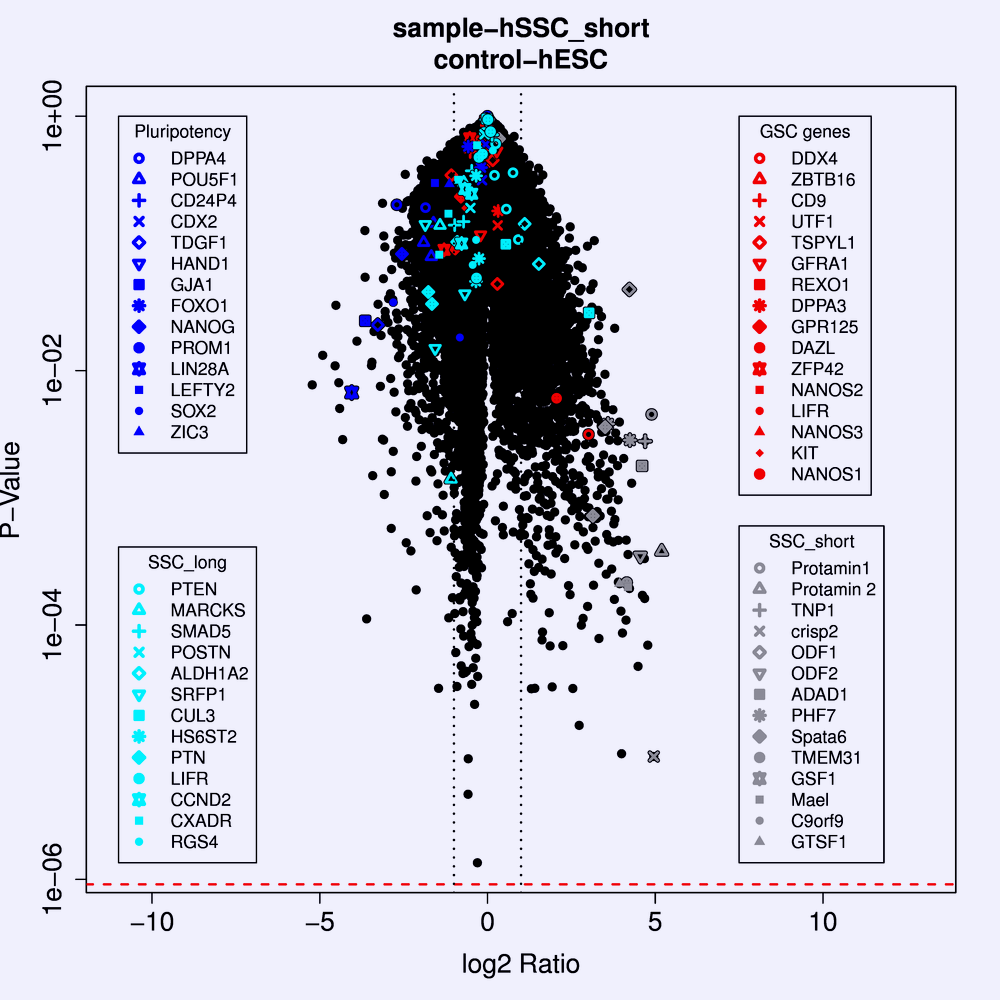

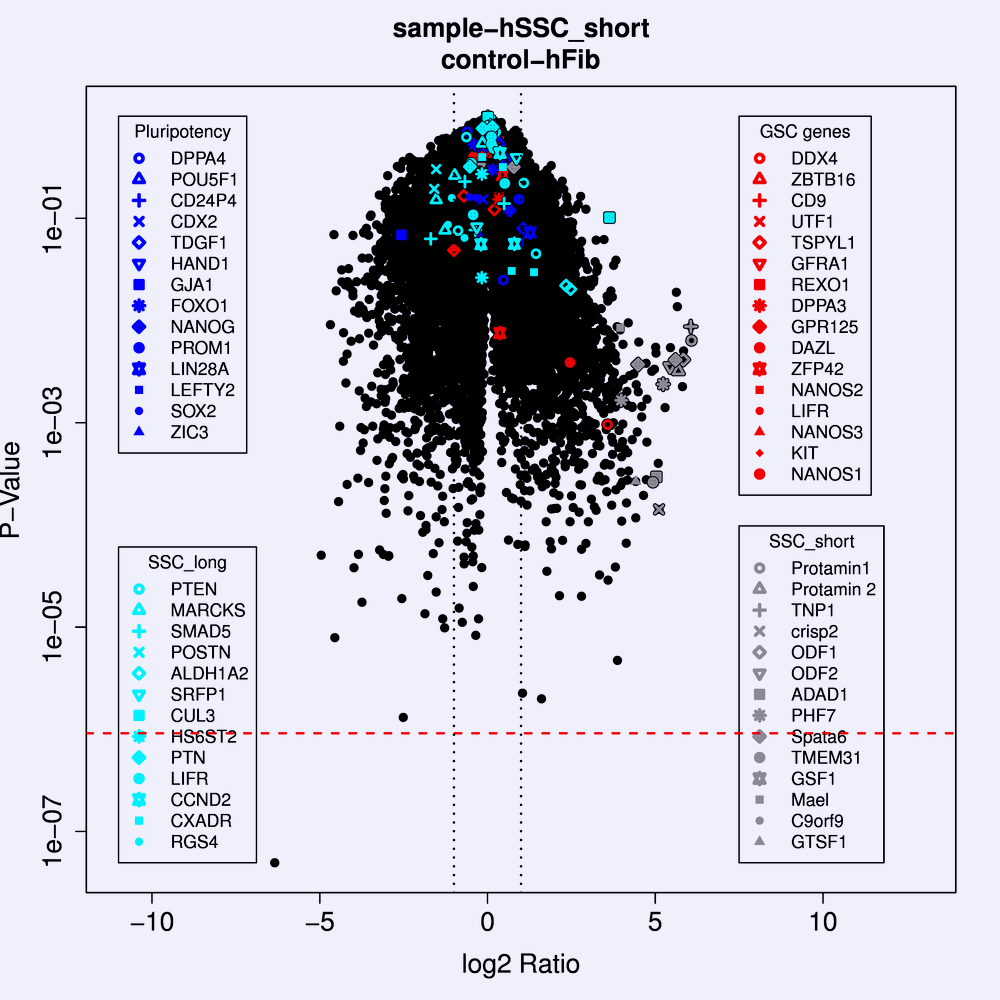

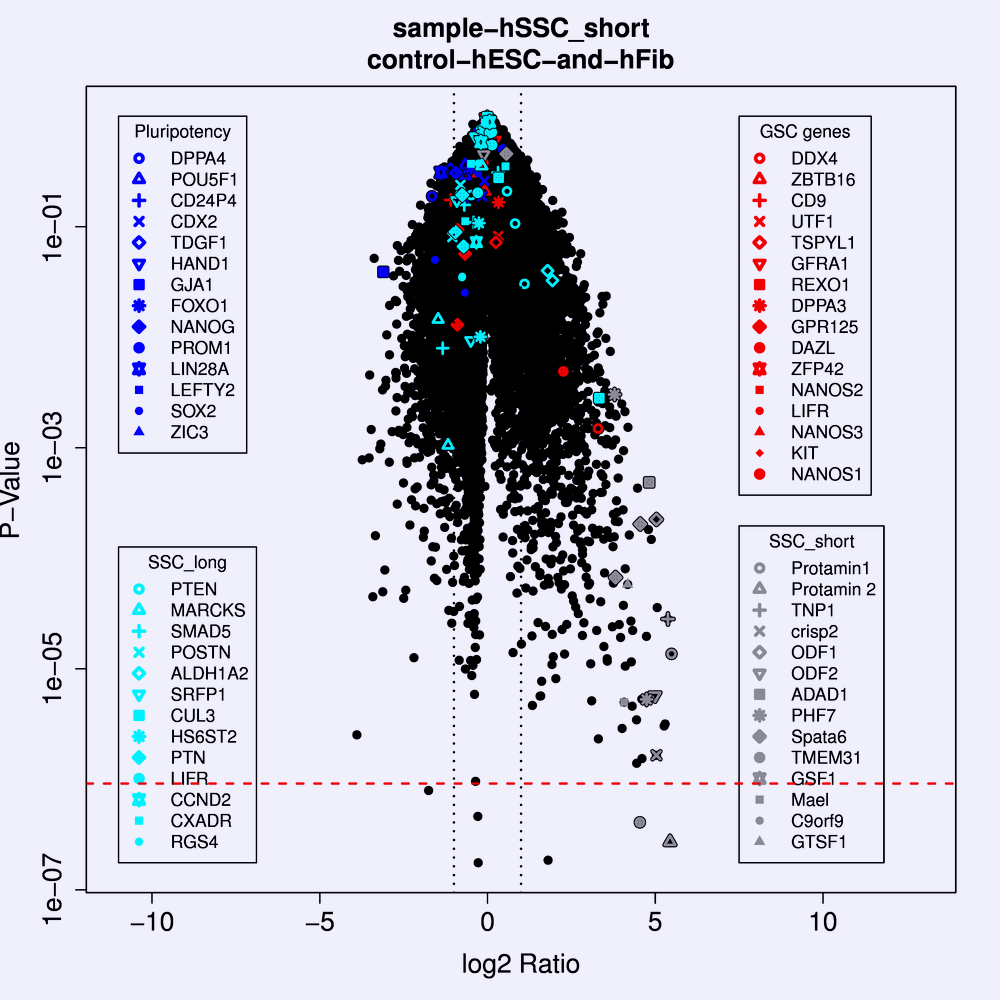
**A B**


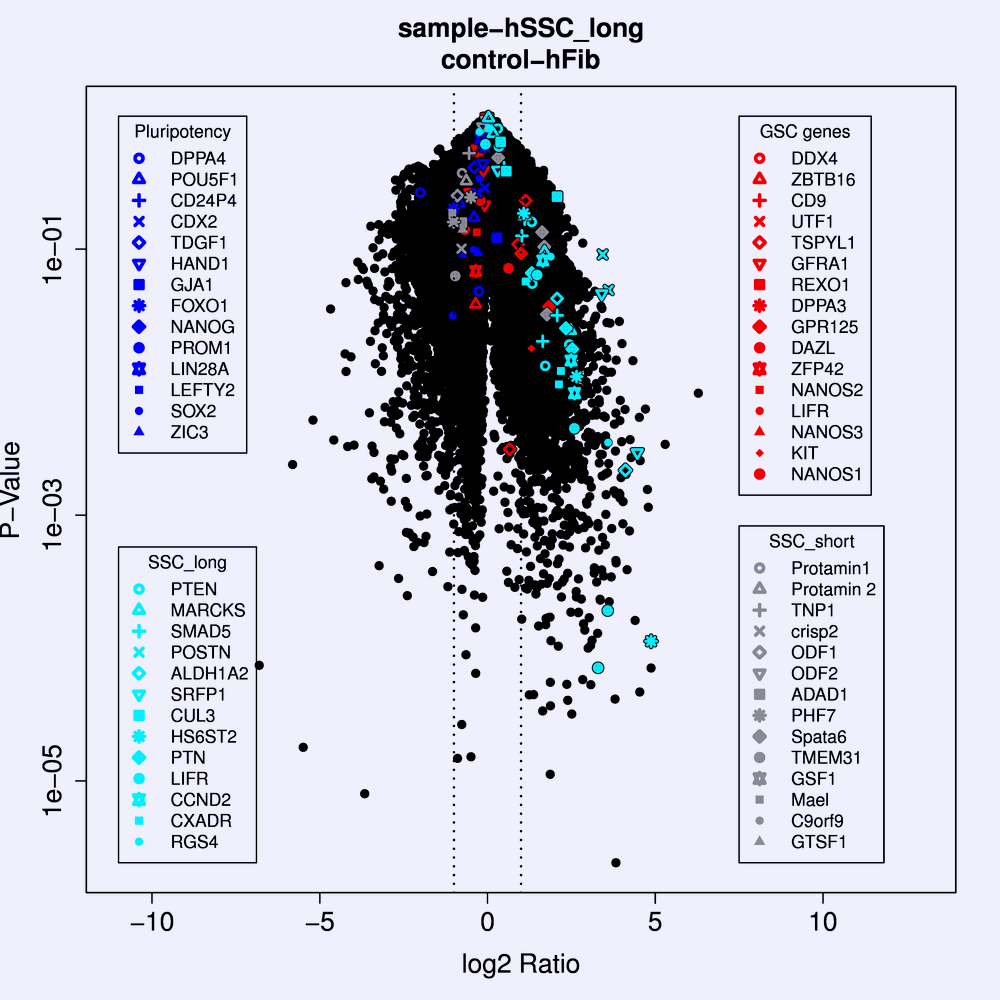

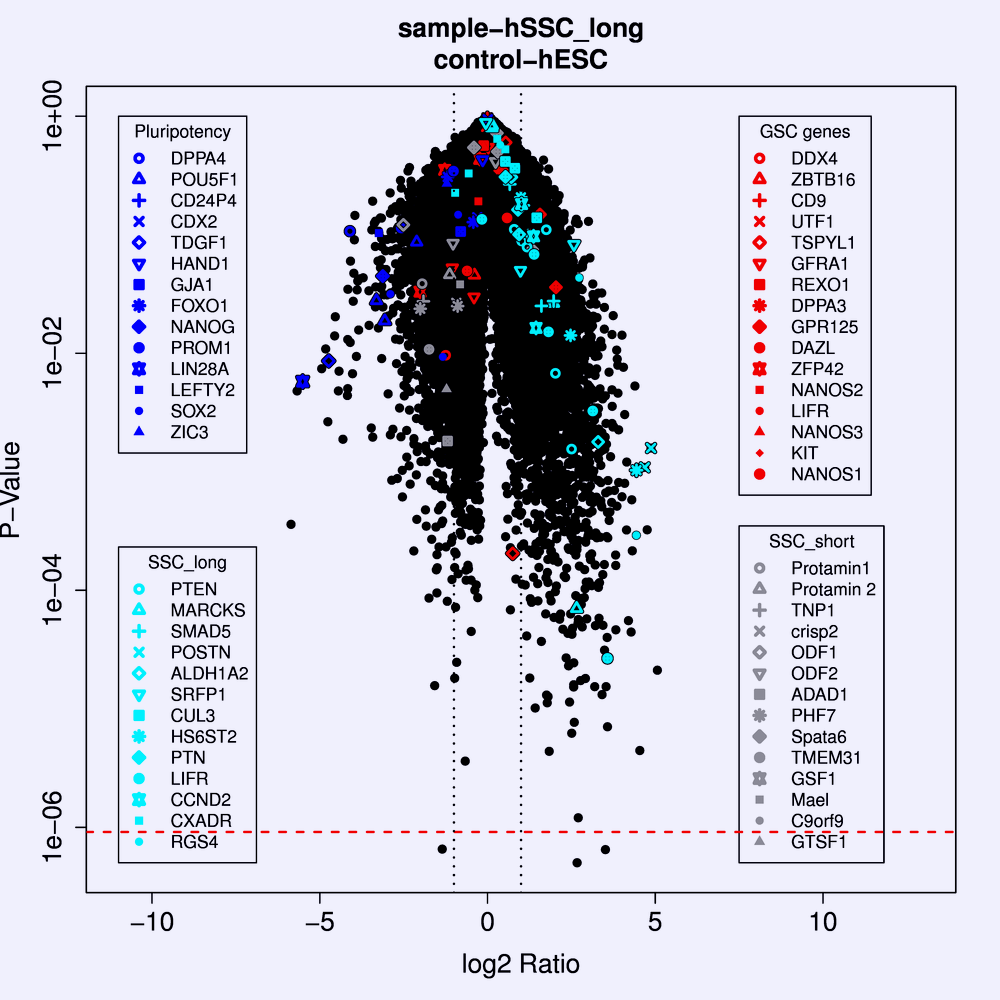

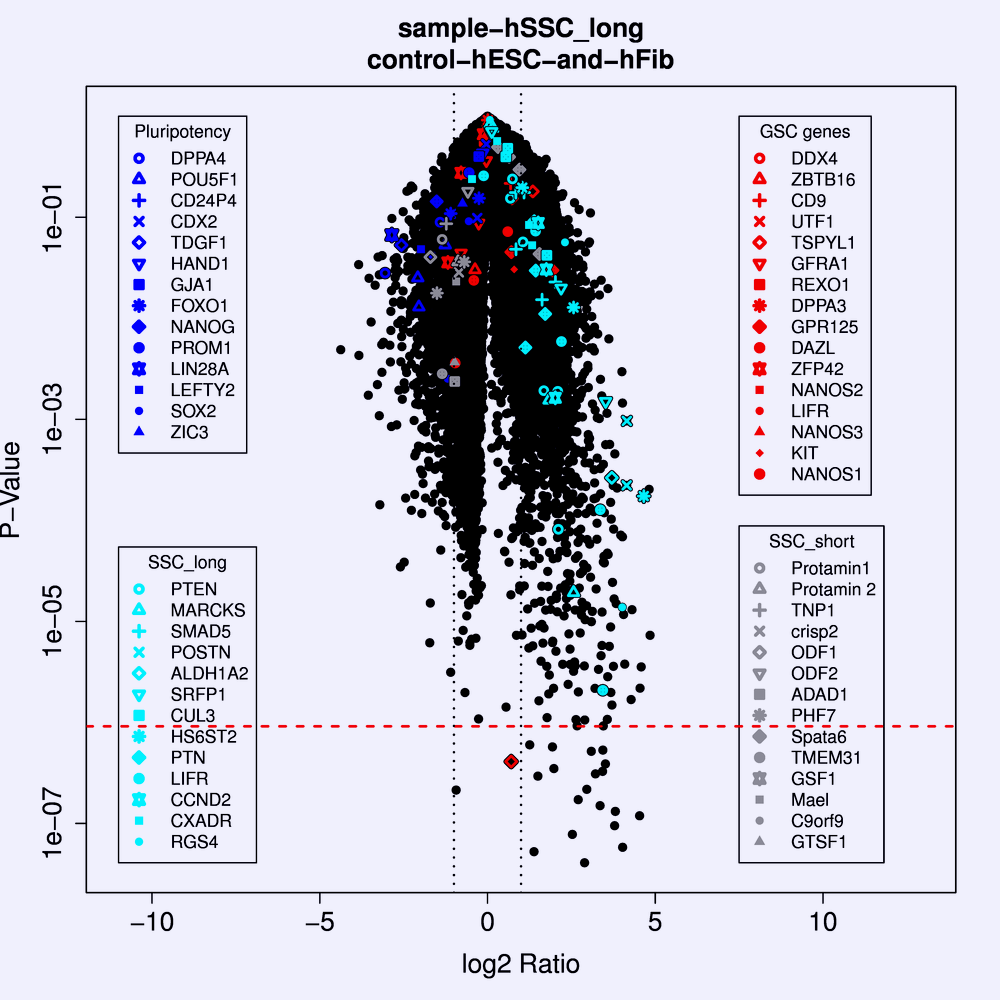

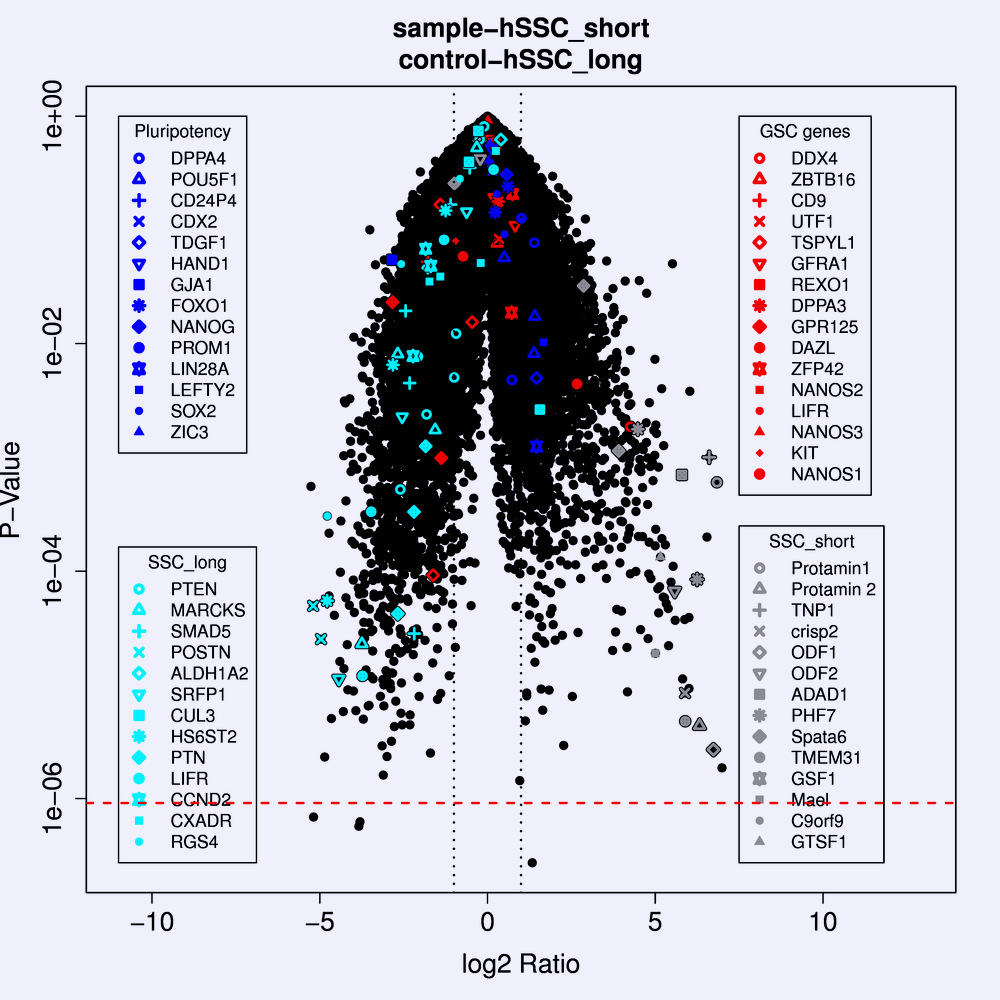
**C D**

**Supplementary Figure 3**

**
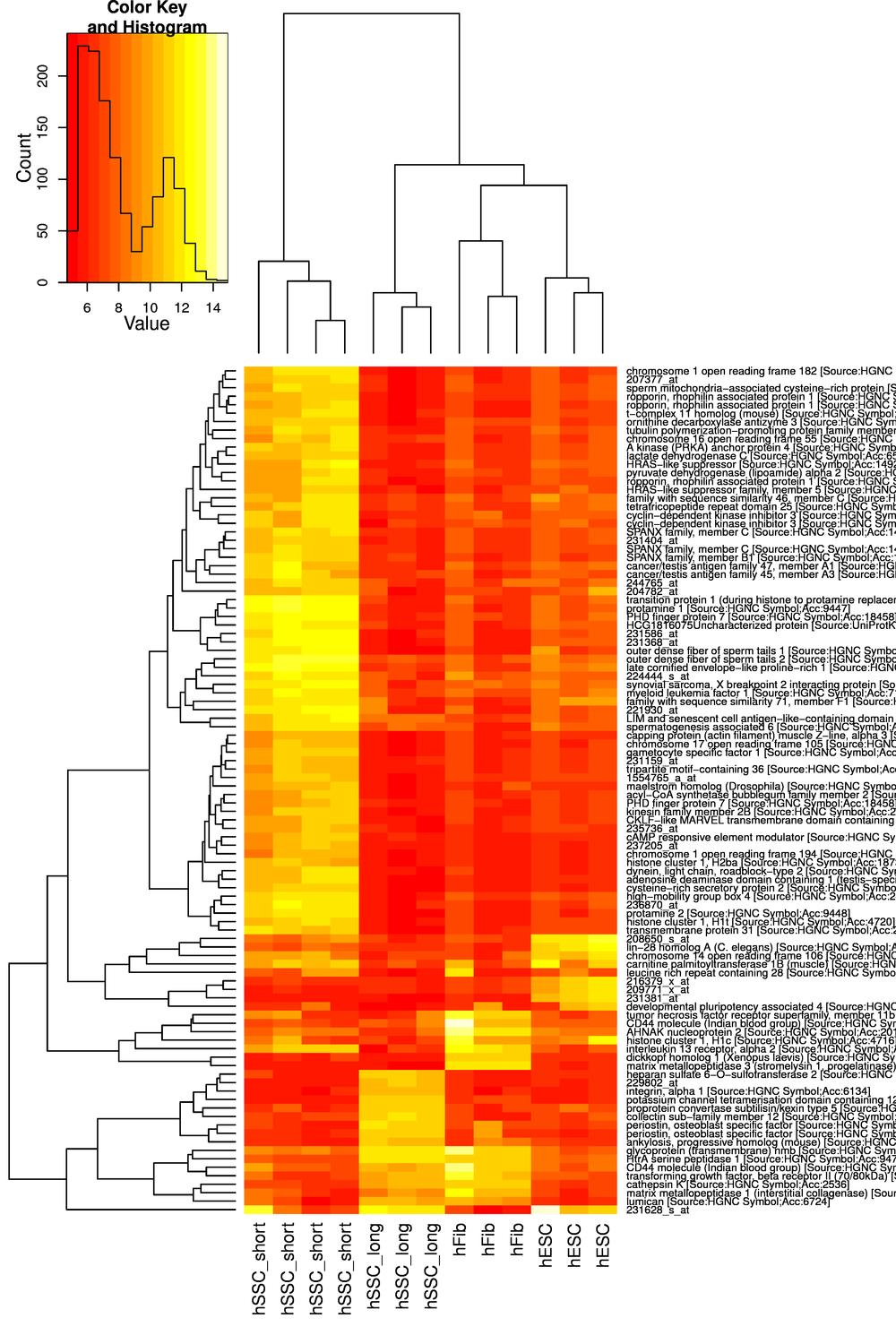
**

**Supplementary Figure 4**

**A**

**
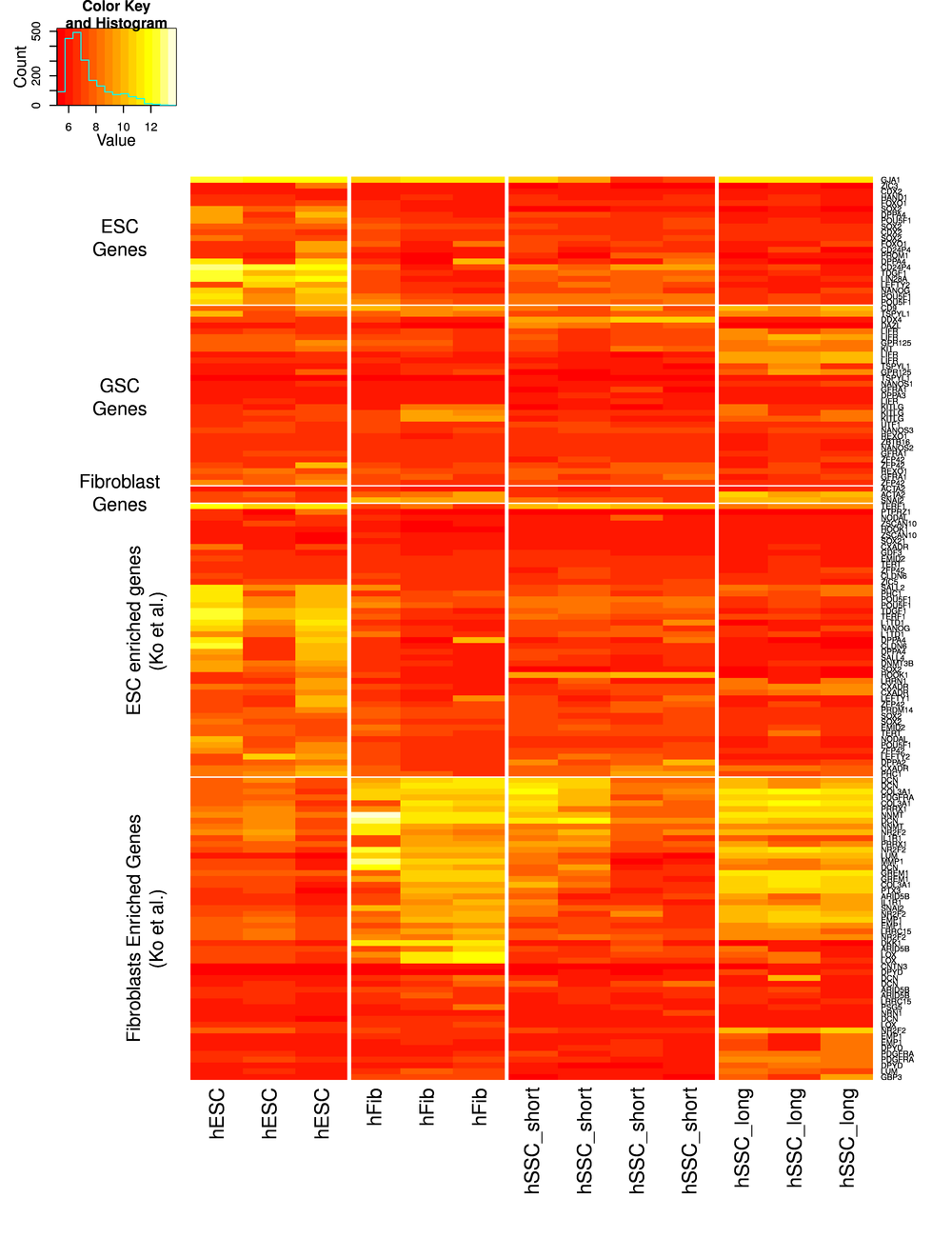
**


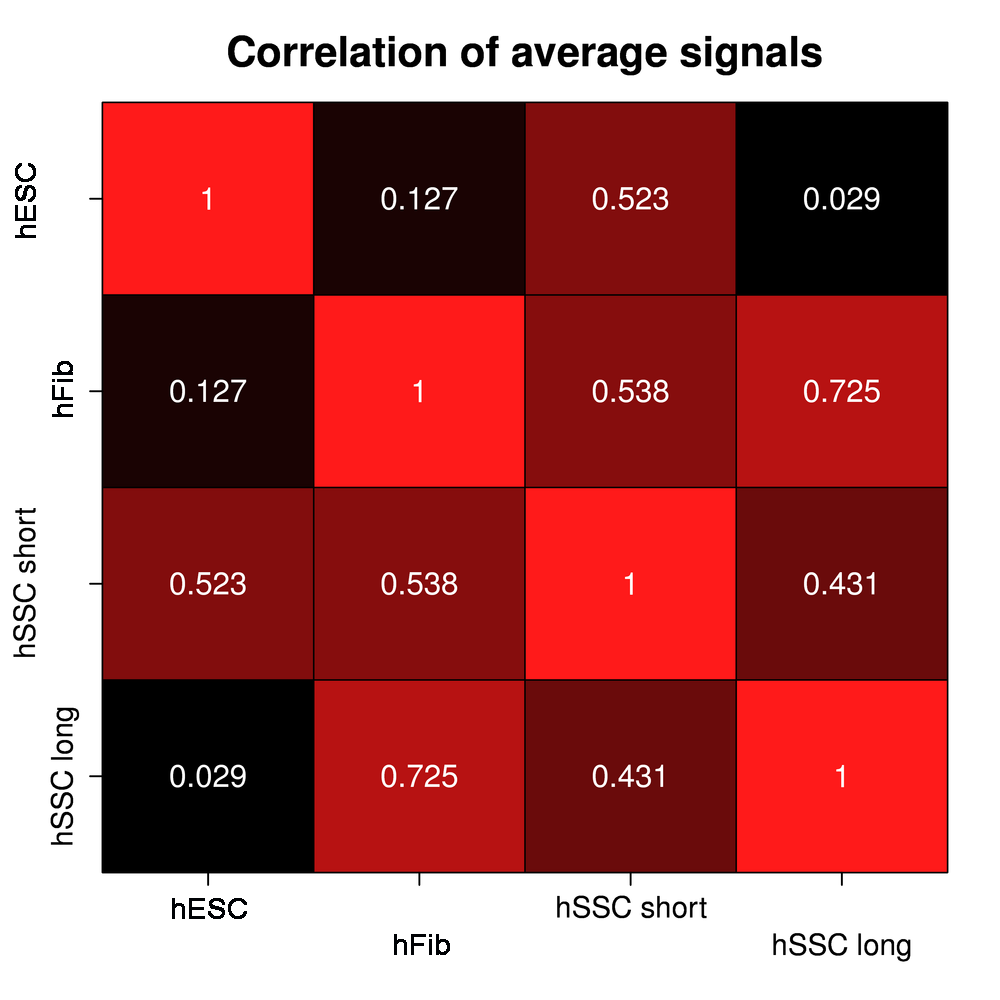
  **B**

**Supplementary Figure 5**

**
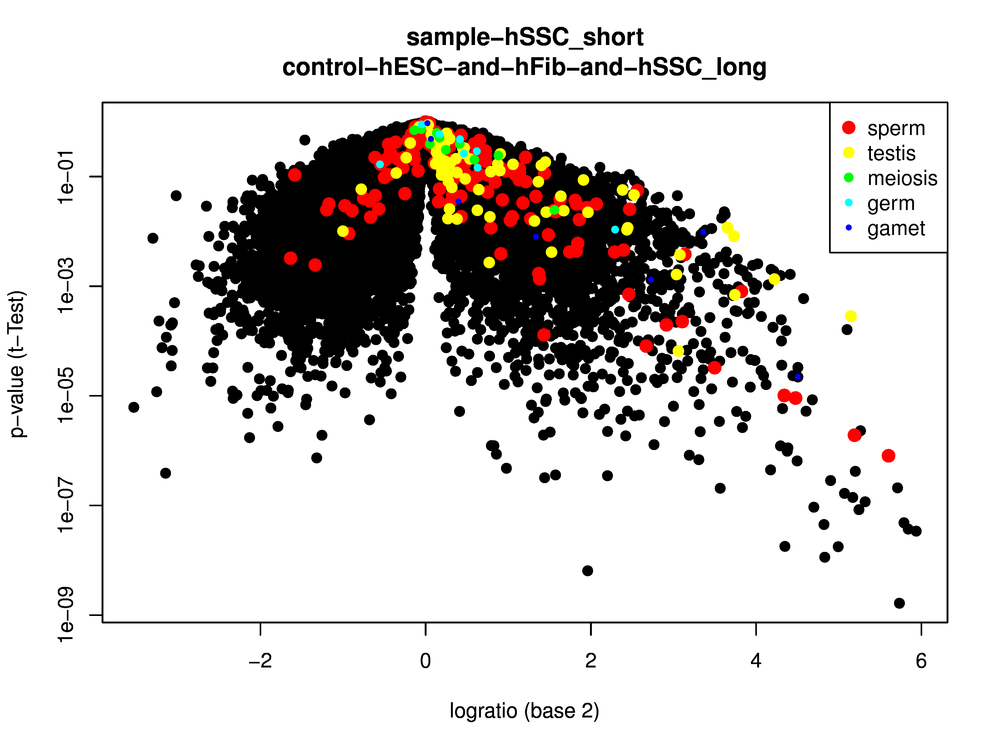
**

.

**Supplementary Figure 6**

**A**


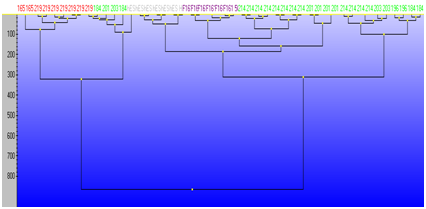
**B**

**C**

**Supplementary Figure 7**


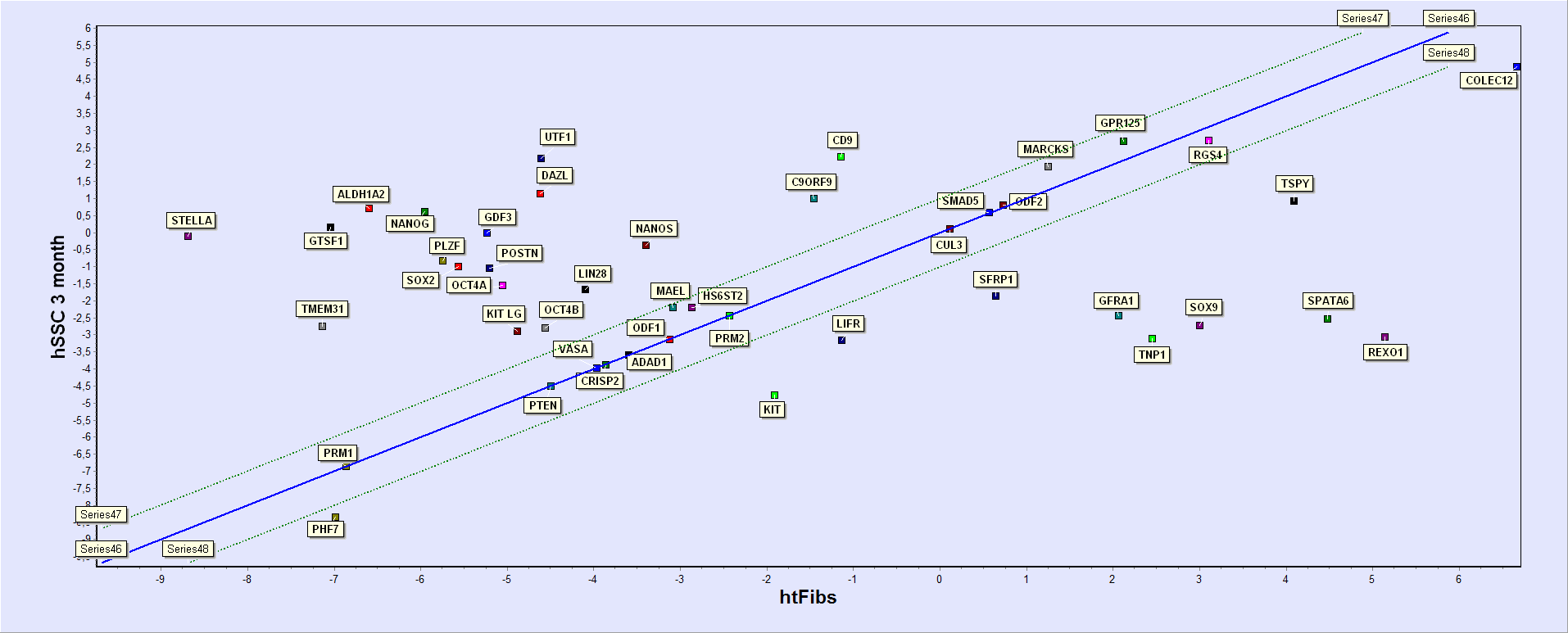

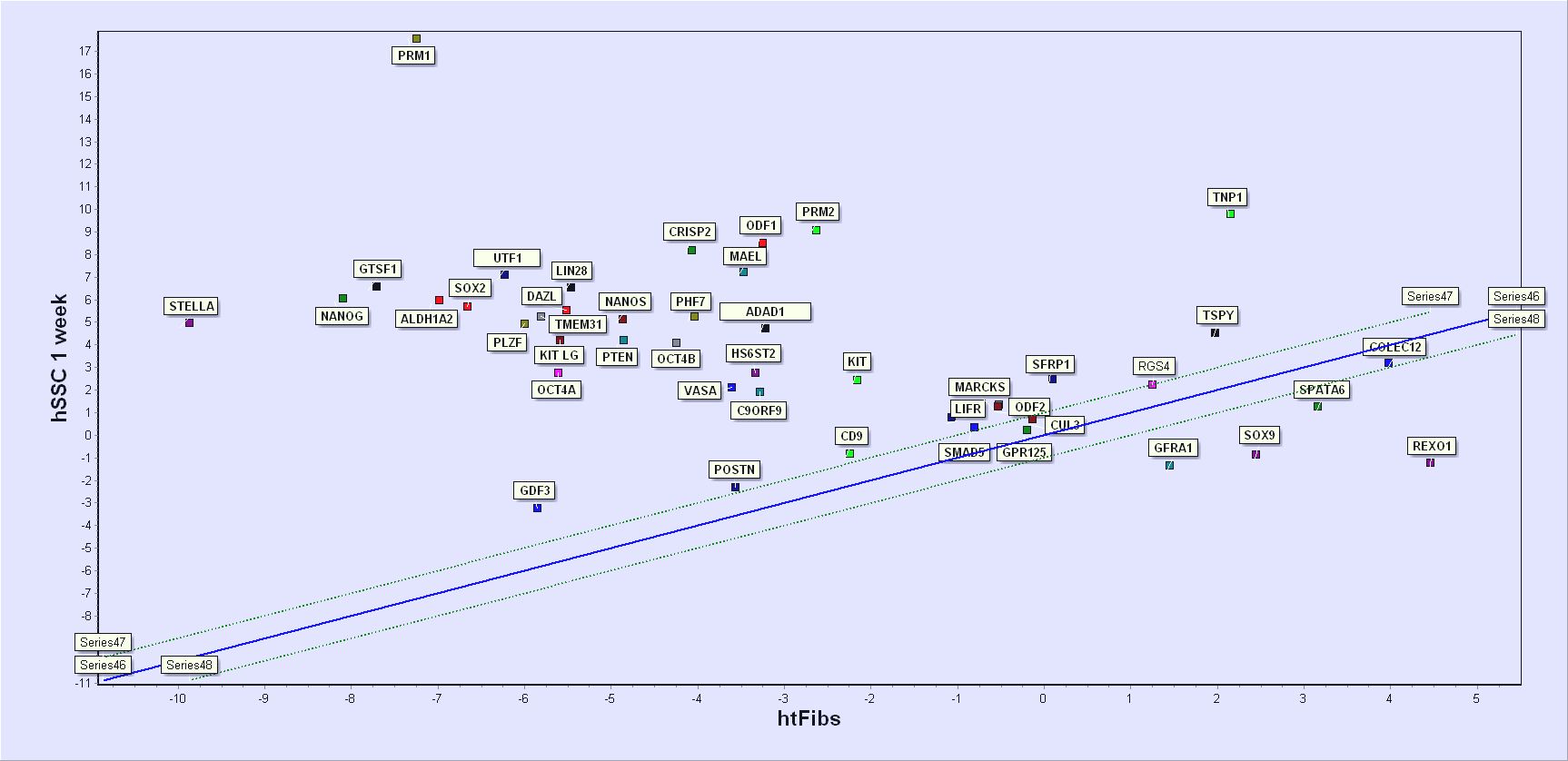
 **A hSSC < 2 weeks - 219 B hSSC 3 month – 214**


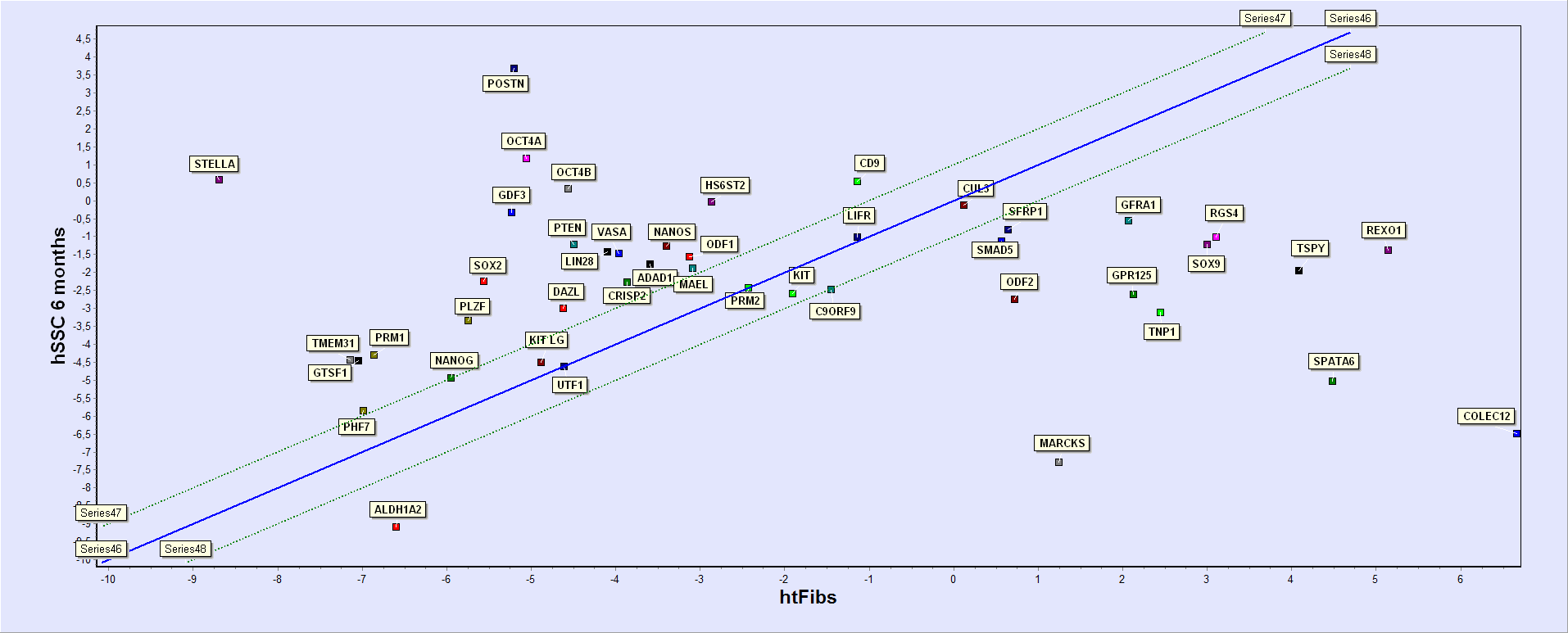

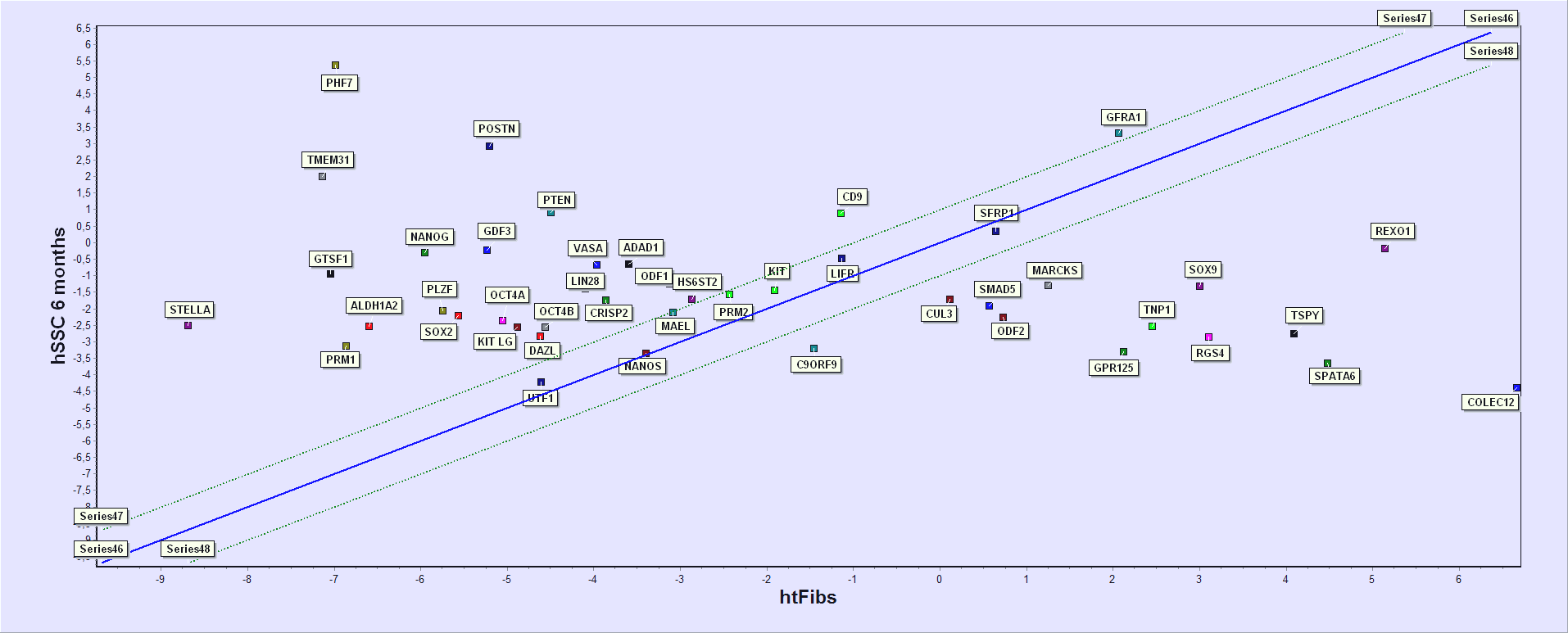
**C hSSC 6 month - 203 D hSSC 6 month – 201**


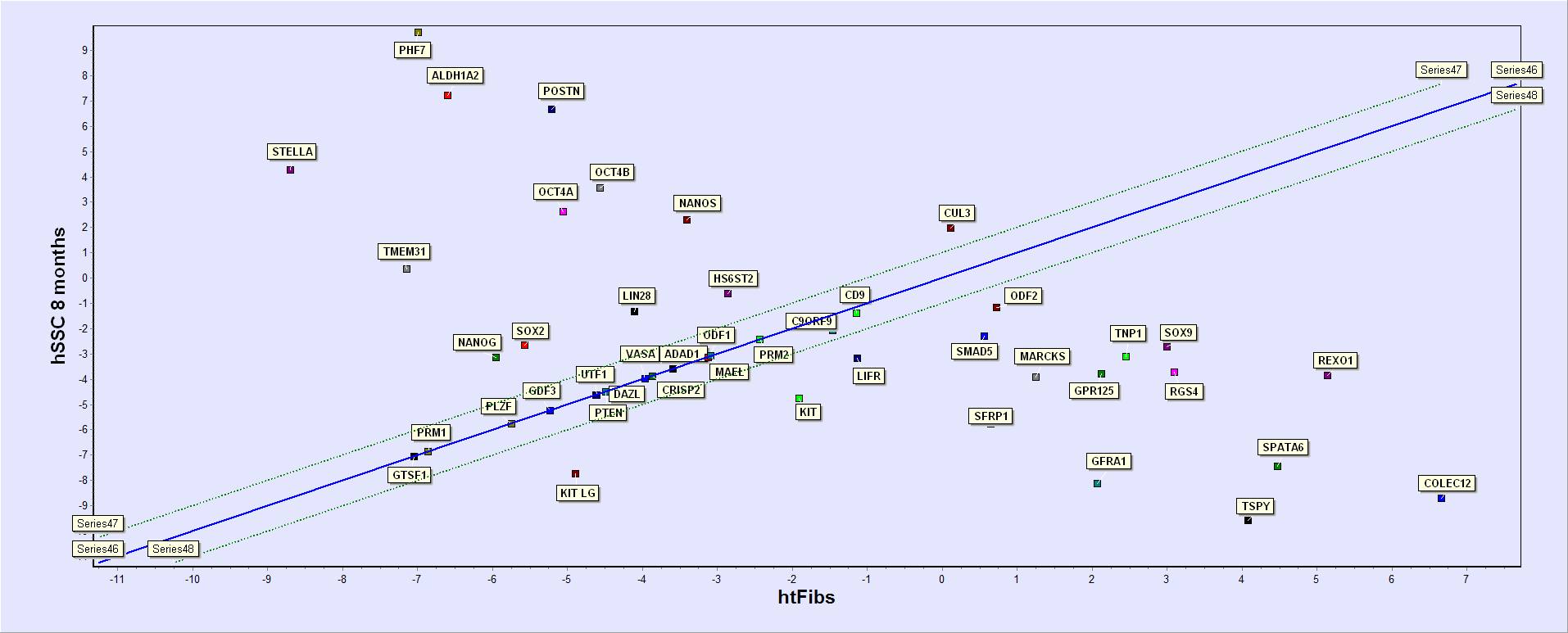

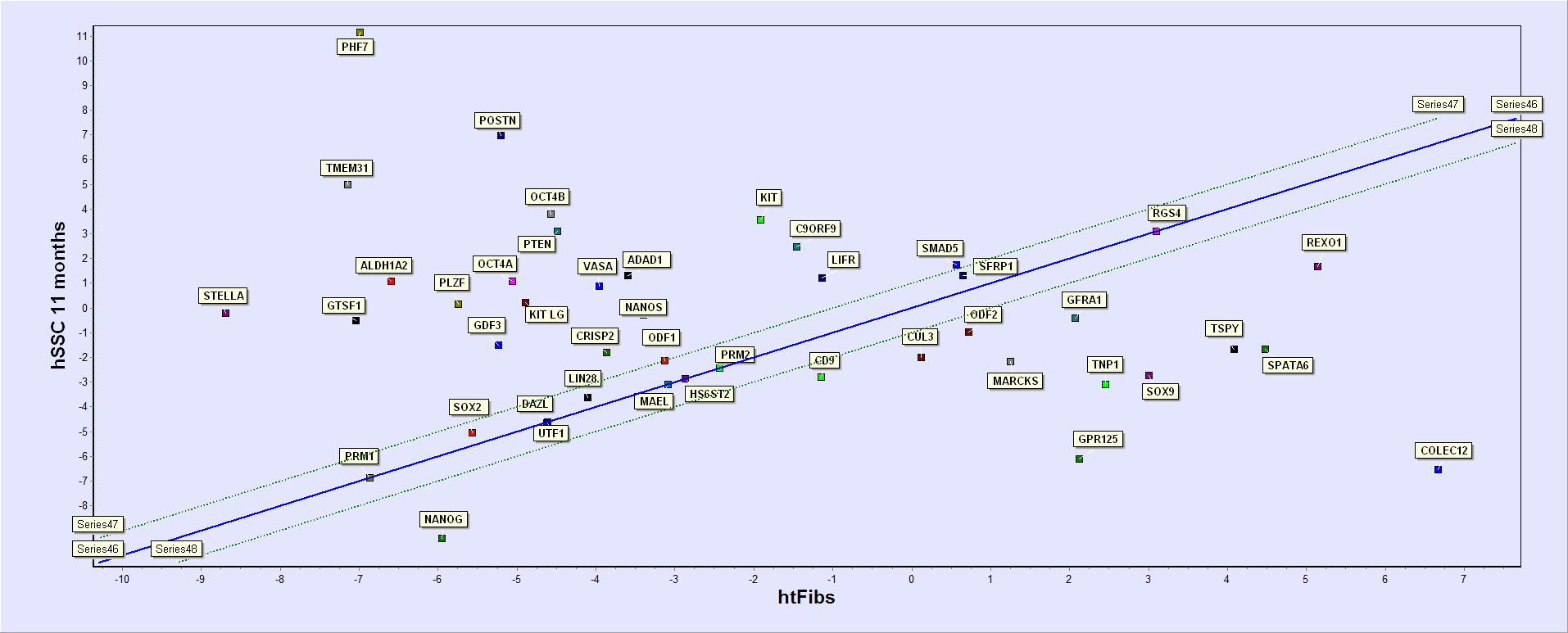
**E hSSC 8 month - 196 F hSSC 11 month – 191**


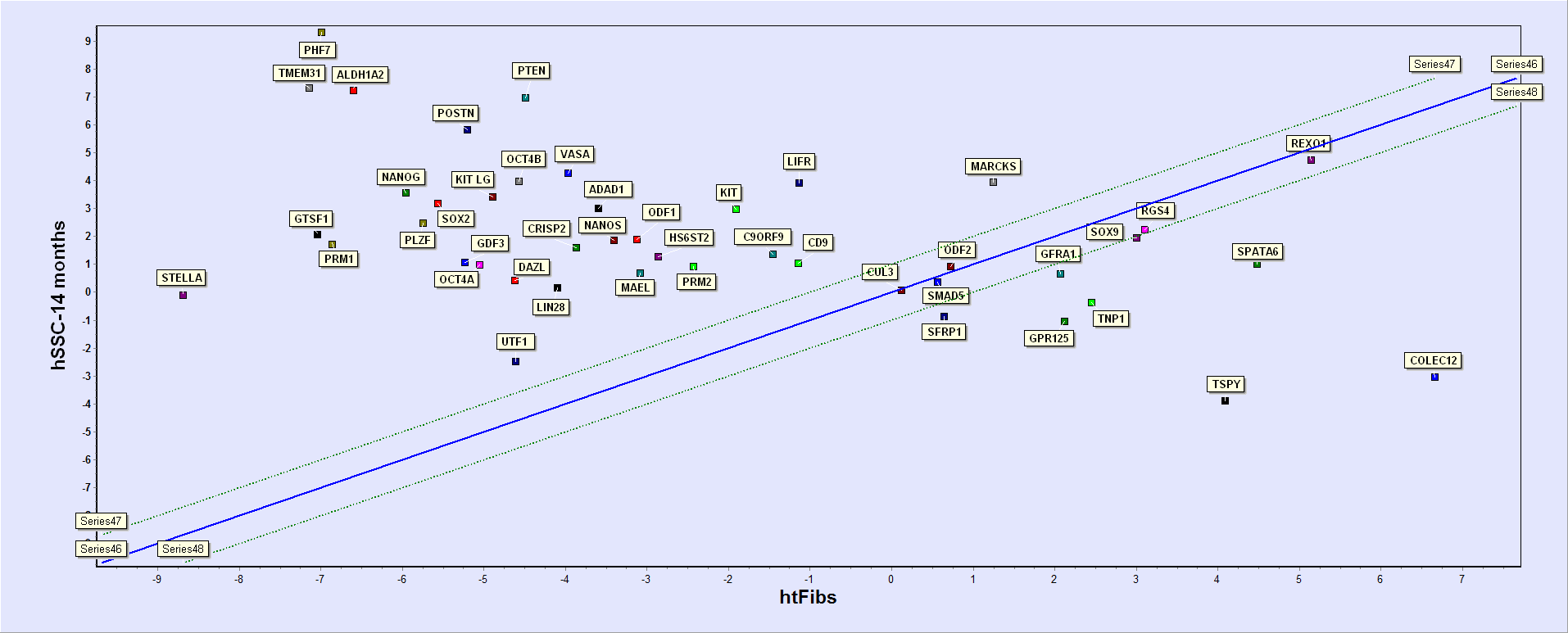
**G hSSC 14 month - 184**

**Suppl. Tables:**

Table s1

| No | Age | Diagnosis | Routine pathological report | Local histology in case of tumor surgery | Date of surgery | Tissue characteristics |
| --- | --- | --- | --- | --- | --- | --- |
| 184 | 74 | Prostate cancer, status after radiatio | Regular maturing, but quantitatively reduced spermatogenesis (hypospermatogenesis), minor tubulussclerosis, no malignancy |  | 23.04.2010 | 2 x testicular parenchyma from bilateral subcapsular orchiectomy, 3.5 ccm each |
| 189 | 45 | **-** | Normal spermatogenesis |  |  | 0.1 g tissue |
| 191 | 28 | Sex reassignment surgery | Arrested spermatogenesis (maturation arrest), congruent to condition of estrogen doses, no malignancy |  | 07.07.2010 | Parenchyma from 2 testes, 3.5 ccm each |
| 194 | 47 | Sex reassignment surgery | Left testicular parenchyma: no spermatogenesis (Sertoli Cell Only Syndrome); right testicular parenchyma: regressive altered testicular parenchyma, significantly reduced spermatogenesis (hypospermatogenesis), both sides: no malignancy |  | 11.08.2010 | Parenchyma from 2 testes, 4 ccm each |
| 195 | 42 | Seminoma right testis, pT1 | No evaluation of peritumoral tissue | Testicular parenchyma with regular matured spermatogenesis, no intratubular germ-cell neoplasia, no malignancy | 02.09.2010 | Peritumoral tesitcular parenchymal biopsy, 1 ccm |
| 196 | 84 | Prostate cancer | Atrophic testicular parenchyma with strong reduction of spermatogenesis (hypospermatogenesis), no malignancy |  | 01.10.2010 | Testicular parenchyma from subcapsular orchiectomy, 5 ccm |
| 201 | 58 | Sex reassignment surgery | Right testicular parenchyma: seminiferous tubules with partial spermiogenesis, several interstitial Leydig cells, interstitial edema, no malignancy |  | 27.10.2010 | Parenchyma from right testis |
| 203 | 20 | Sex reassignment surgery | No spermatogenesis, only limited number of spermatogonia (Sertoli Cell Only Syndrome), definite decrease of Leydig cells, no malignancy |  | 08.12.2010 | Parenchyma from right testis |
| 214 | 54 | Sex reassignment surgery | Right testicular parenchyma: atrophic testicular parenchyma, partly scarred obliterated seminiferous tubules, Focal spermatogonia, predominantly Sertoli cells, only a few residual Leydig cells, no malignancy |  | 23.03.2011 | Parenchyma from right testis |
| 219 | 20 | Sex reassignment surgery | Regular testicular parenchyma, reduced but full matured spermatogenesis, no malignancy |  | 06.06.2011 | Parenchyma from right testis |

**Table S2**

| **Patient/spermatogonia** | **Fluidigm pilot study analyses** | **Microarray**  **Analyses** | **Fluidigm validation**  **of microarray analyses** |
| --- | --- | --- | --- |
| **184** | Long-term culture  (1 month)  Long-term culture  (3 months) | Long-term culture  (6 months) | Long-term culture  (14 months) |
| **189** |  | Short-term culture  (< 2 weeks) |  |
| **191** |  |  | Long-term culture  (11 months) |
| **194** | Long-term culture  (1 month) |  |  |
| **195** | Short-term culture  (< 2 weeks) | Short-term culture  (< 2 weeks) |  |
| **196** |  |  | Long-term culture  (8 months) |
| **201** |  |  | Long-term culture  (6 months) |
| **203** |  |  | Long-term culture  (6 months) |
| **214** |  |  | Long-term culture  (3 months) |
| **219** |  |  | Short-term culture  (< 2 weeks) |

**Table S3:**

**(A)**

**20 most up-regulated genes in short-term cultured spermatogonia versus hESCs, htFibs and long-term cultured spermatogonia**

**20 most up-regulated genes in short-term cultured spermatogonia versus htFibs**

**(B)**

**20 most up-regulated genes in long-term cultured spermatogonia versus hESCs, htFibs and short-term cultured spermatogonia**

**20 most up-regulated genes in long-term cultured spermatogonia versus htFibs**

**(C)**

**Stem (pluripotency)- and germ cell-associated genes up-regulated in (a) short-term cultured and (b) long-term cultured spermatogonia in comparison to hESCs and htFibs.**

**(A)** **Short-term culture of spermatogonia.**

**(B) Long-term culture of spermatogonia.**

**Supplemental Methods**

**Fig. 2.1**


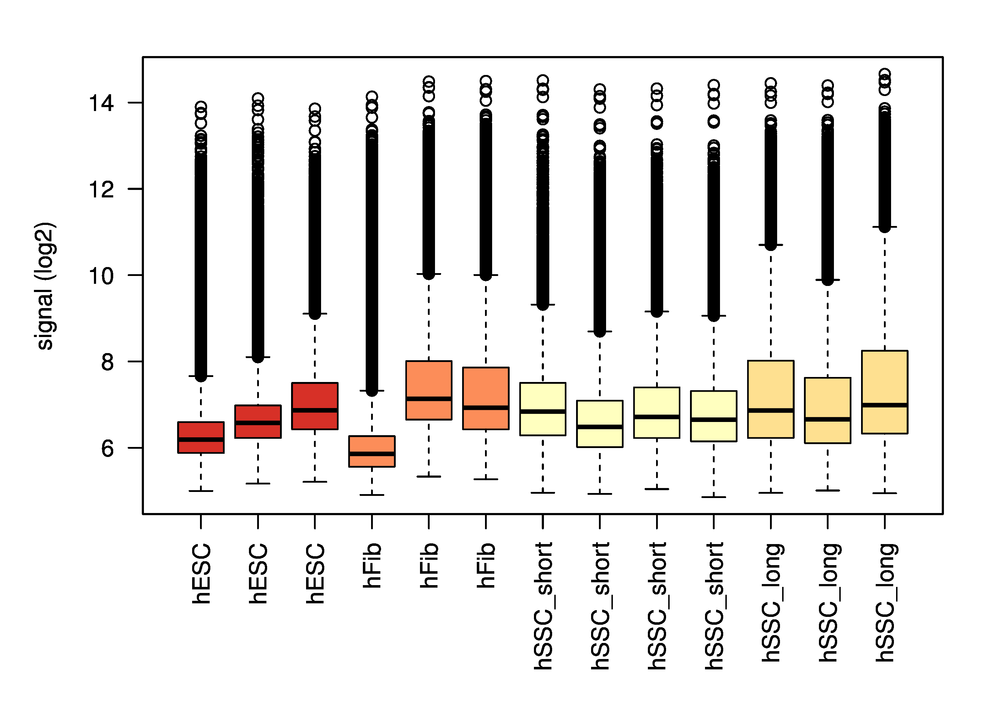


**Figure 2.2**:


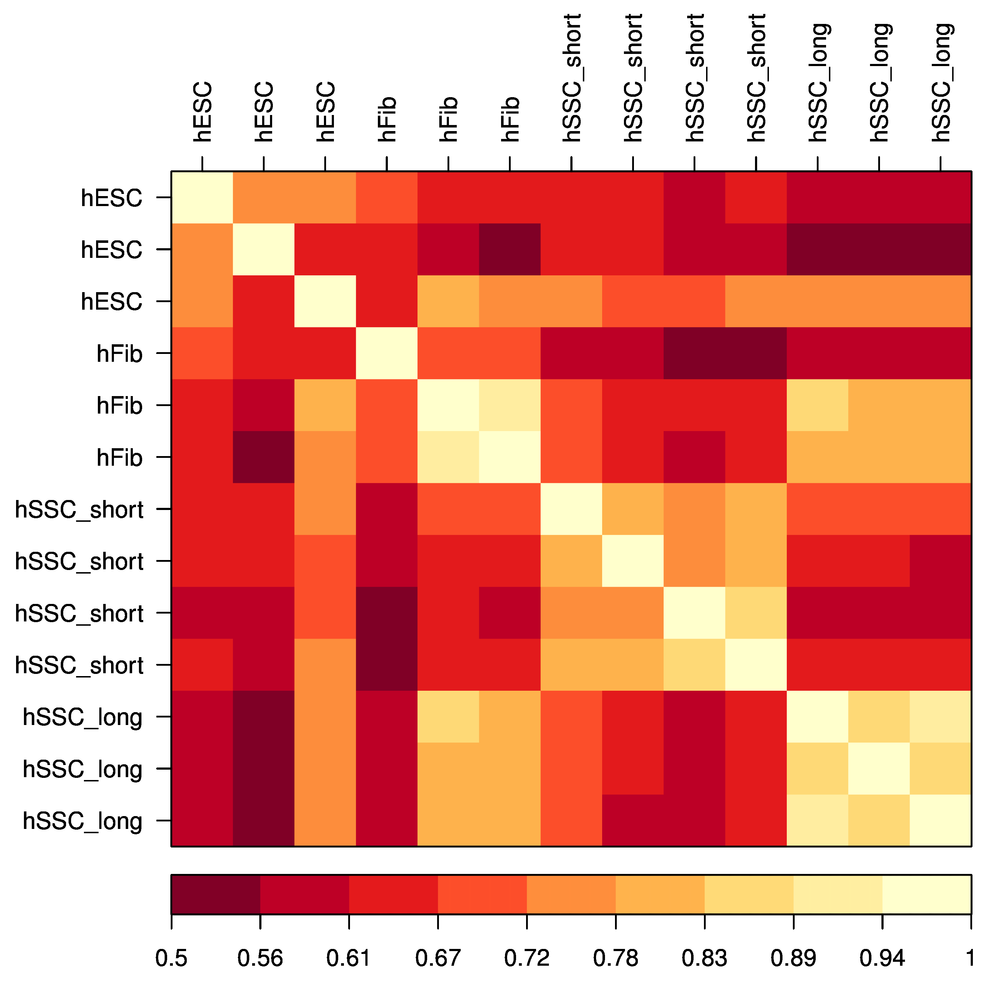


*Multi-lowess normalised data*

**Fig. 2.3**:


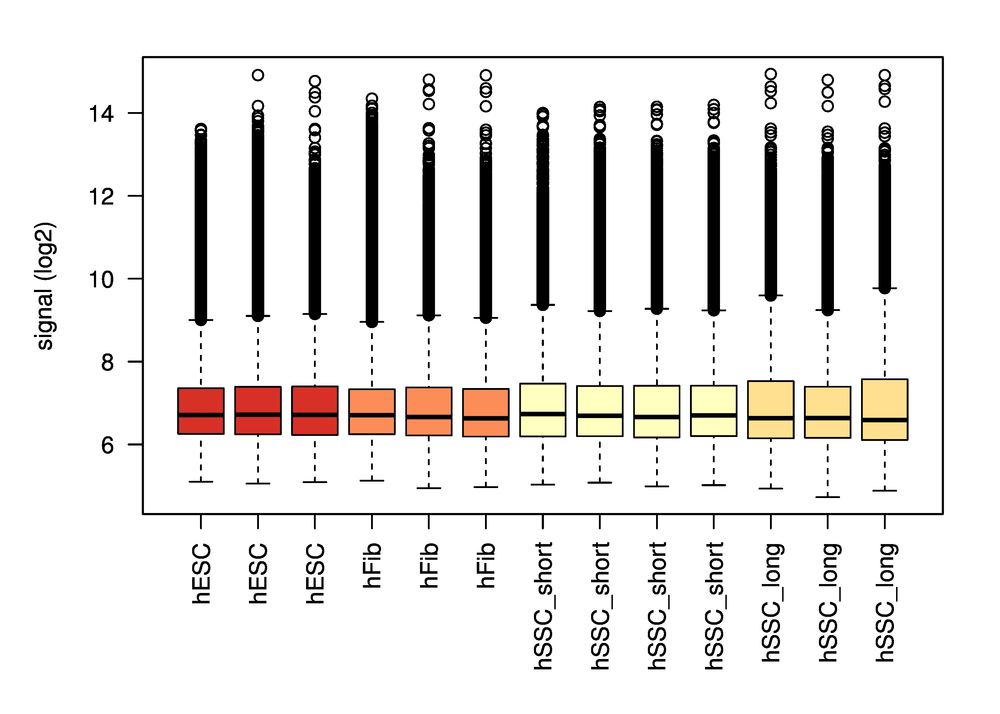


**Fig. 2.4**:


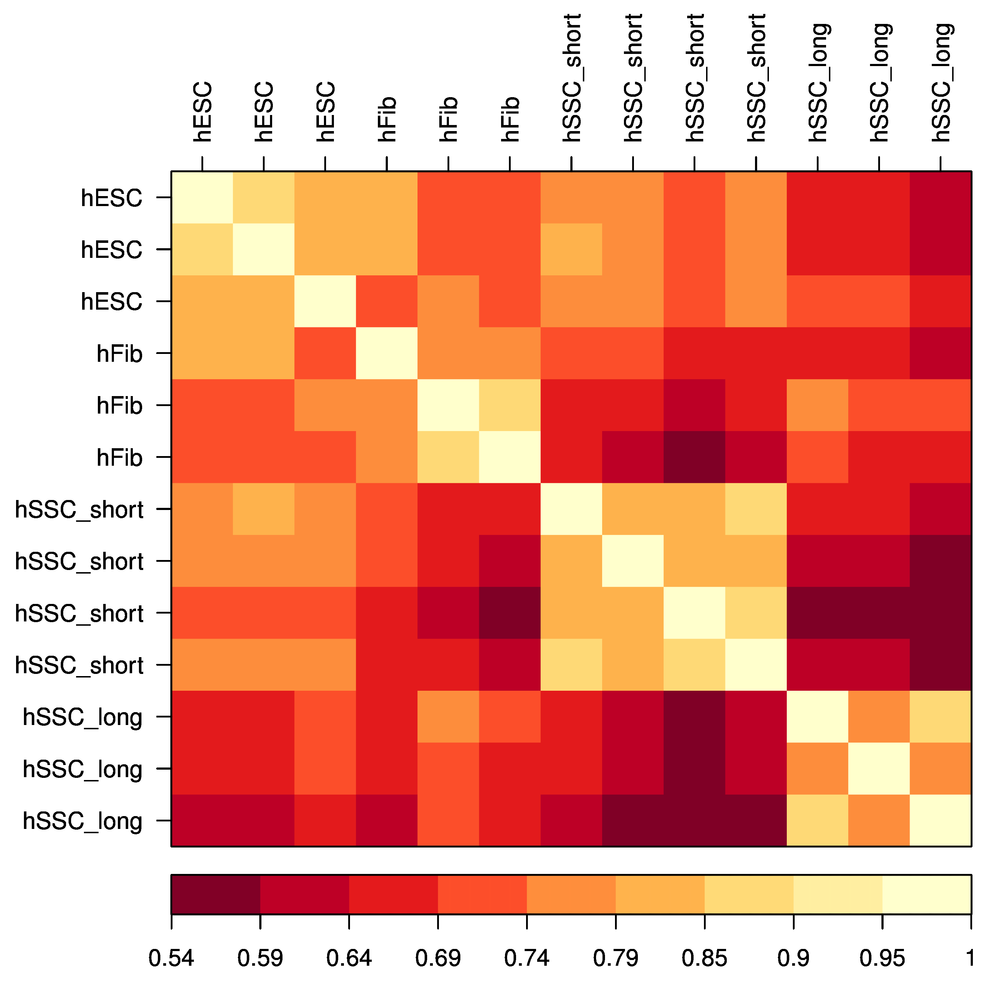


**Figure S1: Germ, pluripotency and fibroblast-related gene expression profiling of samples with 50 spermatogonia from short-term (patient 195) and long-term cultures (patients 184, 194) in comparison with human embryonic stem cells and human testis fibroblasts with Fluidigm Biomark system.**

(**A**) Bar plots with htFibs, hES, hSSC (short term culture < 2 weeks; patient 195), hSSC 1 month (patient 194), hSSC 6 month (patient 184), hSSC 1 long (patients 184 and 194), hSSC all (patients 184, 194, 195). (**B**) Scatter blot analysis of germ-, pluripotency- and fibroblast-enriched genes: (**a**) hES cells, (**b**) short-term cultured spermatogonia (patient 195), (**c**) long-term (1 month) cultured spermatogonia (patient 194), (**d**) long-term (6 months) cultured spermatogonia (patient 184) in comparison with testis fibroblasts.

**Figure S2: Volcano plot (x-axis: log2 ratio and y-axis: t-test p-value).** (**A**) Control cell, hES cells and htFibs (**B**) short-term cultured hSSCs in comparison to hES cells, htFibs, hES and htFibs (**C**) long-term cultured hSSCs in comparison to hES cells, htFibs, hES and htFibs SSC-short. (**D**) Comparison of short- and long-term cultured hSSCs.

**Figure S3: Heatmap of 150 features with the highest variance generated by independent clustering of genes and samples using complete linkage clustering and Euclidean distance.** All cell types are separated consistently and grouped correctly into sub-trees. Each cell type is characterized by a specific set of highly expressed genes (block pattern).

**Figure S4: Gene expressions for the gene sets: ESC genes, GSC genes, Fibroblast genes and ES cell- enriched genes and Fibroblast-enriched genes.**

(A) Heatmap of gene expressions. Samples were ordered according to experimental design. Genes were separated according to the gene sets and ordered by hierarchical clustering within each group (dendrograms not shown). Expression data were not scaled or centred.

(B) Correlation plot (Pearson Correlation Coefficient) of the four different cell types (SSC-short, SSC-long, hES cells and htFibs). Replications for different cell types were averaged. For calculation of correlation only the genes from the five gene sets were used (ESC-specific genes, GSC-specific genes, Fibroblast-specific genes, ES-enriched genes and Fibroblast-enriched genes).

**Figure S5: Volcano plots (x-axis: log2 ratio and y-axis: t-test p-value).** Gene descriptions retrieved from BioMart were parsed for occurrence of pre-defined terms 'sperm', 'testis', 'meiosis', 'germ' and 'gamete'. Genes annotated with 'sperm' (red dots) or 'testis' (yellow dots) were up regulated in short-term cultured spermatogonia compared to control cells

**Figure S6:** **Overall comparisons of the expression of germ and pluripotency genes, characteristic for hSSC in short- and long-term cultured spermatogonia,** **hES cells and htFibs.**

(A) 3D PCA, (B) dendrogram. Group colours in (A) and (B): grey: hESC cells; pink: htFibs; red: short-term cultured spermatogonia; green: long-term cultured spermatogonia. (C) Heatmap with values ranging from strongly expressed (red) to absent (green) with hierarchical clustering are shown.

**Figure S7: Comparison of the expression of germ cell-enriched genes in spermatogonial cell cultures (SSCs) and human testicular fibroblasts (htFibs).** (**A**) hSSCs in a patient 219, < 2 weeks of culture). (**B**) hSSCs in a patient 214, 3 months of culture. (**C**) hSSCs in a patient 203, 6 months of culture. (**D**) hSSCs in a patient 201, 6 months of culture. (**E**) hSSCs in a patient 196, 8 months of culture. (**F**) hSSCs in a patient 191, 11 months of culture. (**G**) hSSCs in a patient 184, 14 months of culture.

**Table S1:** Overview of the patients with age, diagnostics and testicular tissue characterization.

**Table S2:** Overview of the testicular tissue samples used for the pilot-study with Biomark (Fluidigm), the microarray analysis and the Biomark (Fluidigm) validation of microarray data and duration of cell culture at the time of experiment. For validation of the basis germ- and pluripoetency associated gene sets and the selected genes from the microarray analysis, Fluidigm real-time PCR profiling was performed with long-term cultures obtained from 6 different patients (184, 191, 196, 201, 203, 214).

**Table S3:** **Most up-regulated genes in short- and long-term cultured spermatogonia.** (**A**) 20 most up-regulated genes in short-term cultured spermatogonia versus hESCs, htFibs and long-term cultured spermatogonia and 20 most up-regulated genes in short-term cultured spermatogonia versus htFibs. (**B**) 20 most up-regulated genes in long-term cultured spermatogonia versus hESCs, htFibs and short-term cultured spermatogonia and 20 most up-regulated genes in long-term cultured spermatogonia versus htFibs. (**C**) Stem (pluripotency)- and germ cell-associated genes up-regulated in (**a**) short-term cultured and (**b**) long-term cultured spermatogonia in comparison to human hESCs and htFibs.

**Supplemental Methods**

*Microarray data quality / normalisation*

Data condensation was performed with R-2.12.1 (2010-12-16) and the Bioconductor package affy-1.28.0. The condensation criteria were: bg.correct = FALSE, normalize = FALSE, pmcorrect.method = ’pmonly’, summary.method = ’medianpolish’. Raw data in this manuscript are a result of the condensation process and are presented as dual logarithms of the signals. Fig. 2.1 presents the (log2) raw signal distributions of all samples referenced in Table 1.1. Signals are in an reasonable range with a typical shape of box and whiskers for Affymetrix analyses. No outliers are present. The medians of the signals span a range of a signal 1, corresponding to a 2-fold change in expression. Therefore, a normalisation was required.

The correlation plot in Fig. 2.2 displays the correlation coefficients (Pearson) of the (log2) raw signals. Correlation coefficients of 0.5 are very low for standard normalisation approaches (vsn, lowess, quantile regression), because these methods are based on the assumption, that 90% of the signals are not altered, and that is hardly true by systematical changes given in Fig. 2.2. Our decision fell on a multi-lowess normalisation, because we think it effectively removes systematic errors keeping a maximum of the original signal.

**Fig. 2.1**: Boxplot raw data. Signals are in a reasonable range with a typical shape of box and whiskers for Affymetrix analyses. Outliers cannot be detected here.

**Figure 2.2**: Correlation plot of whole chip raw data.

**Fig. 2.3**: Boxplots of multi-lowess normalised data.

Fig. 2.3 presents boxplots of multi-lowess normalised data. Signal distributions of all chips are in the same range. The correlation plot of lowess-normalised data is presented in the Fig. 2.4. Correlations are increased on average compared to raw data (Fig. 2.2). Signal distributions are in the same range - normalised data are more comparable than raw data (Fig. 2.1).

**Fig. 2.4**: The correlation plot of lowess-normalised data.

**GenEx analysis**

Missing data in the Biomark system were assigned a Ct of 999 by the instrument software. These were removed in GenEx. Also Ct’s larger than 25 were removed, since high Ct’s in the Biomark 96x96 microfluidic card are expected to be false positives due to base-line drift or formation of aberrant products. For the pilot study missing data were replaced by Cq 34 and data were normalized with the expression of the reference gene *GAPDH*. Linear quantities were calculated and data were converted to log_2_ scale for analysis. For the experiments validating the microarray data, the effect of setting cut-off to 25 was tested by repeating the analysis with a slightly different cut-off and was found to have negligible effect on the analysis results. Technical repeats were averaged and any remaining missing data were replaced by the highest Cq measured + an offset of 1 for each gene separately. Managing missing data is primarily required for downstream multivariate classification of the data. An offset of 1 corresponds to assigning a concentration to the samples with off-scale Cq values that is half of the lowest concentration measured for a truly positive sample. The magnitude of the offset does not influence p-values calculated with non-parametric methods, which were preferred when there were off-scale data, but has small influence on p-values calculated by t-test and on multivariate classification. In essence, the offset tunes the weight of the off-scale measurement compared to the positive reading; larger offset gives higher weight to the off-scale measurement. We tested the importance of the offset by repeating the analysis using a higher offset up to +4, which corresponds to a concentration of 6% of a truly positive sample, and found negligible effect on the multivariate results. Linear quantities were calculated relative to the sample having lowest expression and data were then converted to log_2_ scale for analysis. Because of the very large and uncorrelated cell to cell variation of genes´ expressions normalization to housekeeping genes is not meaningful. Instead, expression levels were presented “per 50 cell”. Average expression of genes in groups was calculated including 95% confidence interval and groups were compared using 1-way ANOVA (Tukey-Kramer’s pairwise comparison) and unpaired 2-tailed T-Test. Expression of genes with multiple off-scale readings was compared with non-parametric Mann-Whitney’s test. For multivariate analysis to classify samples based on the combined expression of all the genes, data were either mean centred, i.e., subtracting the average expression of each gene, or autoscaled, which is the mean centred data divided by the standard deviation (so called z-score). Autoscaling gives all the genes equal weight in the classification algorithms making them equally essential. Hierarchical clustering (Ward’s Algorithm, Euclidean Distance Measure) including heatmap and principal component analysis (PCA) were performed.
